# Enriching operating room based student learning experience: exploration of factors and development of curricular guidelines

**DOI:** 10.1186/s12909-022-03793-x

**Published:** 2022-10-26

**Authors:** Talat Waseem, Hadia Munir Baig, Rahila Yasmeen, Rehan Ahmad Khan

**Affiliations:** 1 Department of Surgey, Shalamar Medical & Dental College, Lahore, Pakistan; 2 Department of Medical Education, Riphah International University, Islamabad, Pakistan

**Keywords:** Operating room, Operation theater, Learning, Student, Resident, Simulation lab, Quality of learning experience, Structured learning, Structured clinical encounters, Structured surgical encounter template

## Abstract

**Background:**

Operating Room (OR) is a high-pressure setting where multiple complex surgical, educational, and administrative facets interplay. Contrary to resident training, the dynamics of undergraduate medical students’ learning process is highly demanding, opportunistic, unstandardized, and suboptimal owing to many reasons. Upon reviewing the existing published literature regarding the medical students’ experience in the OR setting, it was clear that this field is still to date, unstructured, and ambiguous, with many grey areas that need to be worked on. To achieve an optimized and enhanced theatre experience, it is of immense importance to recognize the recurrent themes affecting medical students within this setting and deduce ways to overcome these challenges. This study explores and prioritizes factors influencing OR-based student learning quality and develops guidelines for structured clinical encounters within the OR setting.

**Methods:**

The study involved an extensive literature review and thematic analysis to generate themes and subthemes, which were subjected to a modified Delphi technique where students and teachers participated to identify, debate, and produce a consensus on the relative value of these factors. Finally, expert-validated guidelines were developed for OR curricular designs*.*

**Results:**

Operating theater-based student learning is multifactorial. Structured learning through optimized course planning, content selection, assessment, and administration are decisive in determining the quality of OR learning experience. The teacher’s interest, attitude, and students’ desire and preparedness to learn play a central role in OR-based student learning, suggesting an enhanced need for adequate faculty training. Similarly, emotional, socio-environmental, and organizational factors can influence students’ learning in a significant way. A new model for undergraduate student learning in OR has been proposed based on these factors and the stakeholders’ interplay. In this model, the teacher’s role is responsible despite OR learning being student- led. Guidelines for the OR curricular designs have been developed.

**Conclusion:**

Structured learning process within the OR setting can lead to optimized lesson planning, content selection, assessment, and administration for a more meaningful and enriched OR learning experience.

**Supplementary Information:**

The online version contains supplementary material available at 10.1186/s12909-022-03793-x.

## Practice points

1. Operating theater-based student learning is multifactorial.
2. Structured learning through optimized course/lesson planning, content selection, assessment, and administration are decisive in determining the quality of OR learning experience.
3. The teacher’s interest, attitude, and students’ desire and preparedness to learn play a central role in OR-based student learning, suggesting an enhanced need for adequate faculty training.
4. Emotional, socio-environmental, and organizational factors can influence students’ learning in a significant way.
5. A new model for undergraduate student learning in the OR has been proposed based on these factors and the stakeholders’ interplay. In this model, the teacher’s role is responsible despite being student-led, and guidelines for OR curricular designs have been developed.

## Introduction

Surgical education, being one of the important components of the medical school curriculum, has traditionally been the focus of scientific debate [1]. Many skills at the undergraduate level e.g. ATLS-related skills, chest tube insertion, knotting, and use of various equipment and instruments though can be well taught in skill labs, still require reinforcement through observation in the Operating Room (OR) environment. Moreover, students find inspiration to opt for surgery as a career when they observe surgery in the OR. Hence, the OR offers students an experience of complex surgical procedures. Most importantly, this setting enhances the learning experience, promotes independent thinking and learning, and facilitates retaining clinical knowledge for potentially improved exam performance [1–3]. Student learning in the OR not only provides undergraduates with exposure to working with real patients but also enables them to adapt to the challenges and stressors related to the OR [2]. The operating room is the most versatile and unique learning environment that focuses on almost all of the learning forms, such as spatial, aural, verbal, physical, logical, interpersonal, and intrapersonal [3]. Moreover, the surgeon’s role is additionally transformed into an educator in the OR, which makes requires more extensive resource and time management skills [4].

Operation theater-based student learning is influenced by a number of factors that likely include emotional, socio-environmental, and organizational factors and factors related to educational relevance and surgical educator [3, 5]. Although a number of studies have individually examined the role of various factors in the OR-based student learning, their relative importance and influence remain partially explored [3]. Moreover, the quality of evidence to substantiate these aspects still remains contextual with low external validity and generalizability [3, 5]. Another critical issue is whether the OR-based learning process should be standardized or opportunistic. Lyon’s model and many others encourage students to apply self-regulated learning to maximize their learning experience in the OR [1, 6]. However, this approach may lead to an unstandardized, opportunistic, and random learning process for medical graduates producing non-uniform student learning and healthcare safety issues. Roberts et al., however, on the contrary, emphasize a more structured approach towards the OR-based learning [7]. There is a need for constructive debate on this issue in the future highlighting whether medical student learning should be structured or opportunistic, teacher-driven or self-regulated, or a combination of both. There are a number of potential learning models which may be of value in operating room environment including Robert’s Briefing-Intraoperative teaching- Debriefing Model, Peyton’s STEP model, 4C/ID model and Lyon’s Model (Table 1 provides a brief overview of the models which are currently being used in the OR setting) [1, 4, 7].

**Table 1:** Brief overview^a^ of commonly used models for teaching and learning in the OR-based environment

Model	Brief Description
Lyon’s Model	The model focuses on the process of intentional learning. It is conceptualized around 3 key domains: the challenge posed by the physical environment; the challenge of an educational task and the challenge of mastering and negotiating the role of a participant in an operating theatre workplace.
Robert’s Briefing-Intraoperative Teaching-Debriefing (BID) Model	The BID model adopts the principles of deliberate practice by focusing both teacher and student on a single goal that guides intraoperative teaching. Intraoperative instruction consists of immediate feedback and guidance guided by specific learning objectives and pre-existing teacher learning scripts. The debriefing element consolidates the learning that occurred in the operation through student reflection.
Peyton’s 4 STEP Model	Peyton’s teaching approach is a step-by-step teaching approach and consists of the following four steps: demonstration, deconstruction, understanding and performance.
4C/ID Model	An ID model is a guide or framework from which an instructional designer creates instructional material or a course. The four components of the 4C/ID model were propagated by Merrienboer and Kirschner (2007). This model has four components: learning tasks, supporting information, procedural information, subtasks.

^a^for detailed review of the models please follow the relevant literature

Similarly, the student and faculty perspective discordance about these factors remain relatively unexplored. This perspective discordance has been discussed in detail in our previous work [8]. Prioritization and building consensus among students and teachers regarding factors influencing OR-based student learning would help formulate guidelines for designing structured clinical encounters within the OR for a more meaningful and enriched learning experience.

The objective of this study is to identify and prioritize the factors influencing (promoting & hampering) in terms of their relative value for undergraduate medical students’ learning in the operating room environment, and to develop guidelines for the effective designing of future OR-based learning curricula to enhance students’ learning experience.

## Methodology

**Fig. 1:**
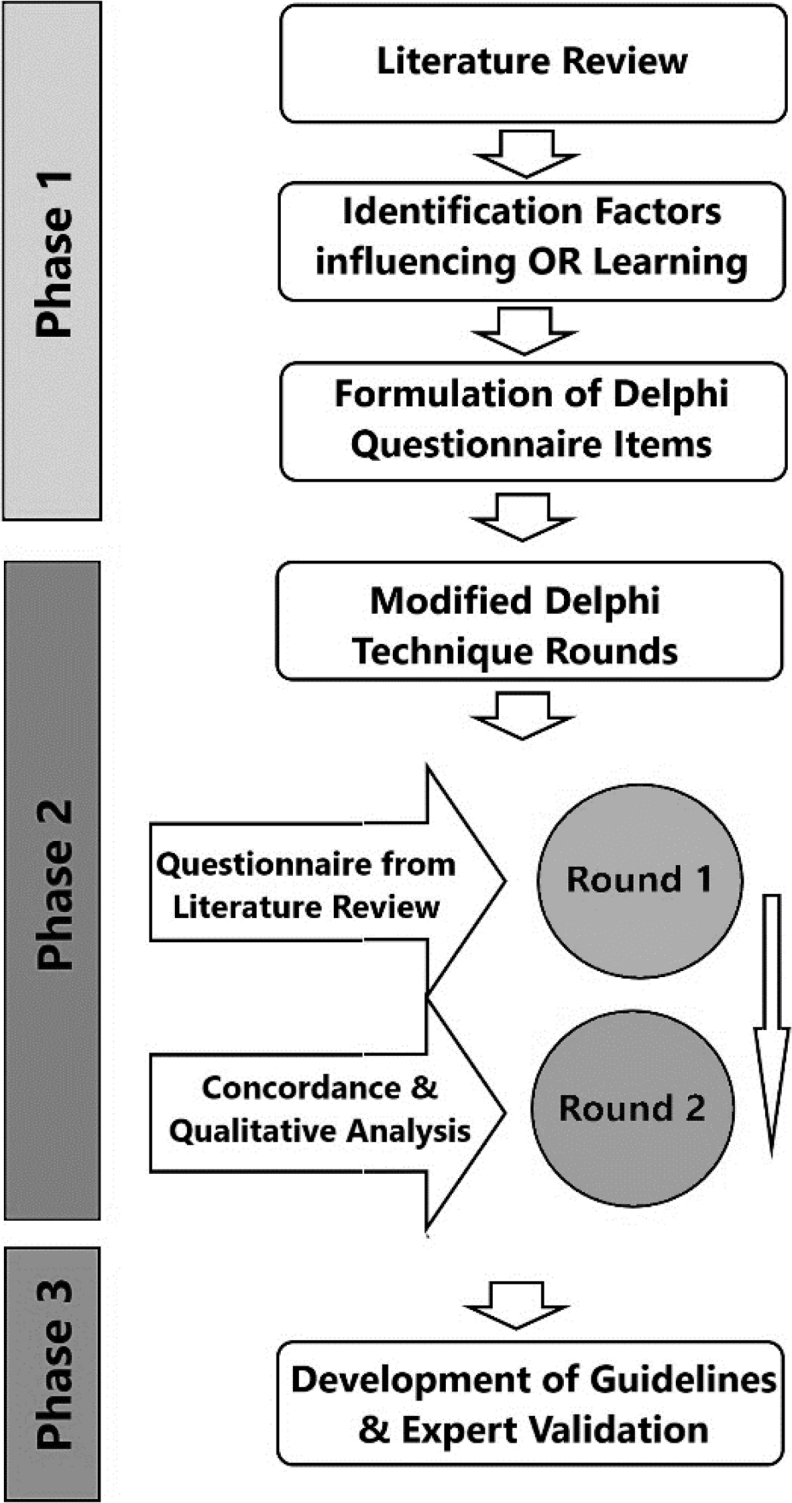
Flowchart explaining the phases of study

Following ethical approval from the University of Lahore’s Ethical Review Committee, this study was conducted between April to June 2020 in accordance with rules and regulations with appropriate informed consenting wherever required. The study consists of three phases, which have been summarized in a flowchart in Fig. 1.

**Fig. 2:**
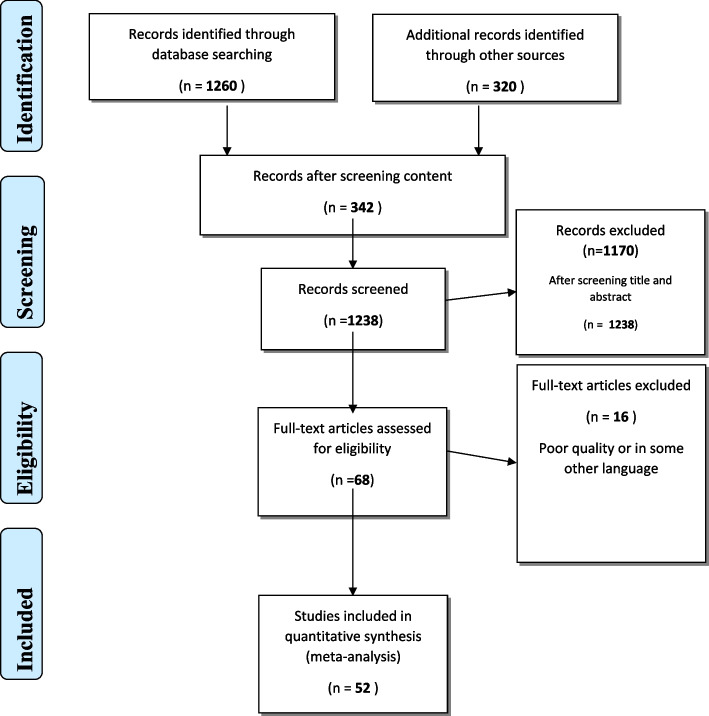
PRISMA Flow Chart for literature search [9]

In Phase 1, following the PRISMA flow chart shown in Fig. 2, the literature search was done through PubMed, ERIC, and Google Scholar using search terms “operating room”, ‘Operation Theater”, “medical students”, “learning”. Thematic analysis and review were performed to identify the factors influencing OR-based student learning. Two authors independently did thematic analysis followed by a collective review for finding the common ground. Based on the literature review themes were ranked according to their relative importance as emerged through the literature review. The themes which were considered un-important or less important were excluded by consensus to limit the scope of the study. Based on the thematic analysis, questionnaire items were prepared for experts and students to be used in Phase 2 attached as Additional file 1: Appendix 1 and Additional file 2: Appendix 2.

In Phase 2, a process of consensus building was opted in form of the Delphi Technique to further elucidate the factors and their relative importance and develop a consensus among the participants. The Delphi method is a consensus-building process in which experts or stakeholders explore a topic or a question for which there is little available information. In an iterative process, they answer questionnaires which are then successively analyzed for themes. Here in this study, we used the Modified Delphi technique. The modified Delphi technique is similar to the full Delphi in terms of procedure (i.e., a series of rounds with selected experts) and intent (i.e., predicting future events and reaching consensus). The main modification is to start the process with a set of carefully selected items [10–13]**.** Based on the Phase 1 literature review items, a modified Delphi questionnaire was developed for Round 1 for both students and teachers. The Delphi technique involved both qualitative and quantitative components. The quantitative component sought the participants’ opinions on a Likert scale for measuring the relative value of the factors influencing students’ OR-based learning. The quantitative component is appropriate for prioritizing the factors, and the Delphi approach itself was quite useful for consensus building. While taking a sample, a purposive sampling technique was used because of the specific nature of the research question [14].

The consensus would be considered achieved once 70% of the participants agree on an issue [10]**.** The qualitative methodology involved the input of suggestions which allowed for an ‘insider’ view of the participants under study. Thematic analysis was done for the qualitative data as described previously.

Quality assurance was established through the maintenance of credibility, dependability, transferability, and conformability. In phase 3, the guidelines were developed, and their content validity and clarity were assessed through expert validation as described previously.

In the Delphi study, participants included surgical teachers with extensive medical education experience and medical students who had attended the surgical rotations. 14 faculty members and 15 students participated in this Modified Delphi Study. In the Delphi study, there are no hard and fast rules about sample size, as suggested previously [11]. Therefore, the decision about panel size was empirical and pragmatic, taking into consideration factors such as time and expense [12]. Representation was assessed by the qualities of the expert panel rather than its numbers [13]. Therefore, in this study, the students and faculty members were purposively sampled to analyze the diversity of opinion so that a meaningful scientific study could be generated. Participants from the University of Lahore, the University of Health Sciences-Lahore, King Edward Medical University, Riphah International Islamic University, Khyber Medical University, University of Dundee, and Queen Elizabeth Hospital Birmingham offering graduation in medicine were included for a diversity of opinion. Majority of the expert panel members belonged to local institutes and were of Asian ethnicity (9/14) two had European and 3 had Caucasian background. Of 14 experts 10 were male and 4 were female. Most of the students had Asian ethnicity (13/15) and out of 15, 12 were male. All of these institutes are tertiary-level institutes with an excellent reputation and structured clinical programs. Faculty and undergraduate students from these institutes participated in this Delphi Study with the eventual aim of designing guidelines for structured learning in operating rooms.

The participants answered the questionnaires distributed through emails in word format. The participants filled out the forms and send them back. The participants were contacted thrice for the responses. The First round had a questionnaire, to which the participants replied and then in the second round they received the Round 1 report and their further input was ascertained. In the second round the participants were also asked to review the proposed guidelines for the curricular design.

Confidentiality was maintained, and participants’ names were not used during any step; participants were given codes for their identity and analysis purposes. All of the participants were blinded from each other. Blinding ensured neutralization of the impact of higher position or influence on the students. The foundations of research were maintained from ontological, epistemological, and methodological perspectives.

Based on this qualitative and quantitative analysis, 3rd Phase of the study focused on developing guidelines for future curricular designs. Two of the authors developed these guidelines and they were circulated among the 14 experts for the item-level content validation (I-CVI). Table 3 describes these consensus guidelines. The expert validation was conducted through the measurement of the item-level content validity index to assess concordance among the experts.

Means were calculated as appropriate. Frequencies, and percentages were counted. Interquartile ranges were calculated as appropriate. The inter-rater reliability was judged through Intra-Class Coefficient as appropriate. Content validity index was measured as described previously.

## Results

The results here would be presented in three phases as per sections of the study.

### Phase 1: literature review on factors affecting student learning in OR

The literature search strategy identified a total of 1580 articles. 52 papers were included after the screening process by removing duplicates. Figure 2 explains the process of literature search and article selection through a PRISMA flow chart. Selected papers were thematically analyzed for finding themes and subthemes affecting students learning in OR. Twenty-one subthemes under four themes were selected, which have been shown in Table 2 in bold. The rest of the themes and subthemes emerged during the Delphi qualitative data input. The literature review’s detailed findings have been published elsewhere [8] and their detailed overview has been omitted in this article to focus more on the Phase 2 findings here.

**Table 2:** Prioritization table of factors influencing OR learning

		Mean Importance Score^a^ on Likert Scale 1–10 Round 1	SD	Mean Importance Score on Likert Scale 1–10 Round 2	SD	% Participant who consider factors either Quite Important or Highly Important	Inter-rater Agreement/ Concordance by Intra-Class Coefficient@ Round 1, Kappa	Inter-rater Agreement/ Concordance by Intra-Class Coefficient Round 2, Kappa
**1**	**Interest of educator**	9.4	0.73	9.463	0.2181	100%	0.881 95%CI (0.794–0.943) *P* = .000	0.892 95%CI (0.827–0.941) *P* = .000
**2**	**Student’s Readiness to participate**			9.219	0.8701	100%		
**3**	**Student’s motivation to learn**	8.89	1.34	9.1675	0.48833	100%		
**4**	**Educator’s behaviour and attitude**	9.06	1	9.1425	0.28077	100%		
**5**	*Number of Students in Batch*			9.125	0.7931	100%		
**6**	**Clarity of Learning Objectives**	9.103	0.97	9.1	0.02	100%		
**7**	**Feeling welcome in OR**	9.03	1.01	9.0878	0.23946	100%		
**8**	**Competency of educator**	9	1.19	9.063	0.2459	100%		
**9**	**Communication of Learning Objectives for OR learning**	9.1	0.87	9.02	0.37	100%		
**10**	**Victimization in OR environment**	8.82	1.2	8.9666	0.40152	100%		
**11**	**Adequate visualization in student learning**	8.89	1.31	8.9419	0.32161	100%		
**12**	**Student’s Focus on Practice of Skills**			8.938	0.84	100%		
**13**	**OR orientation session**	8.86	1.27	8.9131	0.20381	100%		
**14**	**Content Selection**	8.93	1.16	8.8806	0.75501	100%		
**15**	**Teacher’s teaching style**	8.7	1.17	8.788	0.5047	100%		
**16**	*Synchronization of simulation / Lab activities with OR lessons*	8.58	1.15	8.6588	0.47041	100%		
**17**	**Student’s self confidence**	8.69	1.23	8.5919	0.3667	100%		
**18**	**Teacher’s preparedness**	8.379	1.84	8.5819	0.56009	100%		
**19**	**Feasibility of learning objectives to be realistically achievable**	8.62	1.3	8.5594	0.4919	100%		
**20**	**Fear and intimidation in OR learning environment**	8.48	1.66	8.4525	0.33303	100%		
**21**	*Synchronization of the learning objectives with the rest of the teaching.*	8.41	1.7	8.4084	0.40958	100%		
**22**	*Student’s Prior Knowledge*			8.375	1.008	100%		
**23**	**Environmental readiness OR as learning Hub**	8.414	1.42	8.369	0.1768	100%		
**24**	*Student’s self-review of study material*			8.063	0.9817	100%		
**25**	**Anxiety of student in OR environment**	7.89	1.49	7.9488	0.54496	100%		
**26**	**Student’s Personal Learning Objectives in OR Learning**	6.62	1.5	7.0006	1.06305	100%		

^a^Importance Score: 0–2(Not Important); 3–4(Neutral); 5–6(Somewhat Important); 7–8(Quite Important);9–10(Highly Important)

^b^Percentage of the participants who consider these factors as either ‘Quite Important” or ‘Highly Important”

@ Inter-rater agreement / concordance measured by the Intra-Class Correlation Coefficient. (0.8–9.0 = Strong)

### Phase 2: modified Delphi study results

The study participants were both faculty members with experience in teaching surgery and medical education and students who had undergone rotation in the surgery department. The participants belonged to diverse institutes, including local as well as the UK institutes. The teachers’ average age was 43.78 ± 9.30 with a mean teaching experience of 12.64 ± 10.98 and experience in medical education of 5.78 ± 3.8. The students’ mean age was 24.53 ± 1.12.

#### Relative importance of factors affecting student OR learning—quantitative component

The participants evaluated the relative importance of factors identified from the literature review through quantitative analysis of Delphi Rounds 1 and 2. The importance was scored on a scale 0–10 (0–2 = Unimportant; 2.1–4 = Neutral; 4.1–6 = Somewhat Important; 6.1–8 = Quite Important; 8.1–10 = Highly Important). The factors affecting students’ OR-based learning, discussed here in this study, have been rated as either ‘Quite Important’ or ‘Highly Important.’ None were rated unimportant. The factors have been prioritized based on scoring across 2 rounds of the Delphi technique. The inter-rater reliability measured through Intra-Class Coefficient has been estimated to be 0.89, 95%CI (0.827–0.941), which is a strong inter-rater consensus (Table 2). The prioritization table (Table 1) clearly shows that teachers’ interest and students’ motivation and preparedness are the two most important determinants of students’ OR learning quality. The newly emerged themes and sub-themes (5 items, shown in *italic* here) from Round 1 have been incorporated into the list of factors.

#### Relative importance of factors affecting student OR learning—qualitative component

The qualitative analysis of emerging themes, subthemes, and axial codes through the Delphi Rounds was done and is presented in Additional file 5: Appendix 5. Here we describe only the conclusions drawn from this analysis. The details of the final axial codes, themes, subthemes, and representative statements can be reviewed in Additional file 5: Appendix 5.

The participants here clearly argue for more deliberate learning within the OR environment than traditional opportunistic learning. Students and teachers agree that graduates are more likely to benefit from standardized learning than self-driven learning, which may not accomplish all the learning objectives that a standardized graduate should cover. Moreover, newer themes and subthemes emerged about the use of various models of learning in the OR setting. Robert’s BID model and Peyton’s STEP models were favored both by the teachers and the students. The participants agreed on exploring the value of the 4C/ID model in a simulated environment setting. They valued Lyon’s model’s potential role in training the students to cope with the OR environment and maximize their learning in this realm.

The content selection should be shared between the teacher and the student and should focus on all cognitive, psychomotor, and affective domains of learning. The content and education should be synchronized with simulation-based lab work, virtual patients, and standardized patient activities. The learning objectives should be clear, practical, and achievable within the OR learning environmental framework and communicated timely. As described commonly in medical education, they should be *s*pecific, *m*easurable, *a*chievable, *r*elevant, *t*argeted, and *t*ime-bound (SMARTT). The initial orientation session should focus on all relevant aspects of the operating theater, its staff, OR protocols & its working. It should provide a detailed framework of the whole rotation, methodologies of teachings being used, modes of assessment, and interaction/synchronization with the skill lab work.

The teachers have a dominant role within the OR setting. Their interest, competence, attitude, quality of feedback, and encouragement positively influence OR-based learning. Fear, intimidation, and bullying negatively affect student learning and should be discouraged. Teachers and staff’s welcoming attitude has a positive impact. Considering the rate-limiting role of the teachers within the OR setting, it remains imperative to focus on faculty training.

Likewise, students’ motivation and desire to learn, preparedness, and skill to use self-regulated learning can influence their quality of learning experience. The organization needs to support infrastructure and adequate visual and skill lab support to improve the learning experience in the OR.

### Phase 3: Development & Expert Validation of guidelines

Based on this qualitative and quantitative analysis, 3rd Phase of the study focused on developing guidelines for future curricular designs. Table 3 describes these consensus guidelines. The expert validation was conducted through the measurement of the item- level content validity index (I-CVI). Table 3 describes the guidelines and their item-level content validity index agreed upon by the experts. These guidelines provide basic principles that must be kept in mind while designing the OR-based curricula.

**Table 3:** Guidelines for operating room curricular designs

	Guidelines for Operating Room Curricular Designs	I-CVI
**1**	Structured learning positively influences student learning within the OR environment. The learning within OR-based environment should be structured- *not opportunistic*. There should be collaborative content selection by the teacher and student, keeping in sight the required skill set for a standardized graduate.	1
**2**	The teacher’s interest, preparedness for lessons, teaching competence, teaching style, behaviour with students, and a welcoming attitude positively influence the quality of student’s OR-based learning. Teacher training for all these attributes is a pre-requisite for quality OR-based learning.	1
**3**	The learning objectives of OR-based learning should be clear, practical, synchronized with concomitant teaching and need to be defined and circulated prior to sessions in an effective manner. The learning objectives should be developed by the teacher/s with student input for essential components of standardized OR-based learning required for graduation. However, the students should be given room in curriculum to follow his or her own personal learning objectives for enhanced learning at individual level.	**0.931034**
**4**	An orientation session about the theatre complex, staff, teaching provisions, theatre working, basic concepts of sterilization and biomedical ethics can alleviate student’s anxiety and nervousness. An orientation session at the commencement of OR rotation can be quite effective.	1
**5**	Priming students with pertinent educational material prior to lessons, improves student’s participation and understanding. Provide students with relevant educational cognitive material for OR learning prior to OR lessons.	**0.931034**
**6**	Constructive and prompt feedback positively influences student’s OR learning. The teachers should be trained to provide constructive and prompt feedback. Assessment should involve cognitive, psychomotor, and affective aspects of the OR-based learning.	1
**7**	Training to handle OR environment through affective skill training is important for student’s optimal participation in OR lessons. The students should be oriented and trained to overcome their anxieties to better participate within dynamic OR environment.	**0.965517**
**8**	Bullying, intimidation, victimization, and harsh attitude negatively affect the student’s participation in OR learning. Train your faculty to avoid it and ensure a mechanism for accountability.	1
**9**	Student’s ability to effectively interact with theatre staff, motivation and desire to learn and ability to self-regulate learning during OR sessions is pivotal. The teacher needs to ensure that students interact adequately and remain motivated for lessons.	1
**10**	Adequate and individualized interaction is key to enhanced OR-based learning. To improve teacher-student interaction and individual attention, keep student number rotating in OR to a minimum.	1
**11**	The theatre complex should be transformed into a learning hub for medical students, with enhanced visualization of procedures through technology (e.g., LED Screens and microscopes etc.) where ever appropriate, improved synchronization with a simulation lab and better administration.	1
**12**	Motivate and provide opportunities for students to practice skills through well-coordinated skill lab and OR-based psychomotor activities.	1

## Discussion

Teaching and learning in the OR setting have always remained challenging both for the teachers and the students. Medical students have different perspectives and challenges in their learning process as opposed to residents in surgical training who significantly rely on self-regulated and self-paced learning based on principles of adult learning. Therefore, the models and activities that we use for resident training may not be entirely appropriate for medical students. Medical students are generally more dependent on teachers in terms of content selection and less on administration, and they favor more structured and deliberate forms of learning. The scope of this scientific work is to partially evaluate which model or models are appropriate for medical graduates’ learning within the operating room; whether we should rely on a structured learning framework or an opportunistic one or a combination of both; and how we can manage the identified factors influencing OR-based learning from this study in an opted learning framework.

In line with Roberts et al., this study finds that students prefer a more structured learning plan [7]. They feel that teachers can more appropriately choose learning objectives corresponding to the expected skill set required by a graduate, with more experience in this field and having gone through this experience themselves beforehand. Moreover, they are more comprehensively exposed to various aspects of medical learning and patient care and are likely to make better decisions in favor of them. However, they would like to participate in selecting modalities to be used for achieving these learning objectives. There is an increased need to prepare standardized graduates that can function optimally anywhere in the world and provide uniform healthcare.

However, one of the drawbacks of standardization is the potential loss of diversity in medical education. Personal learning objectives are an essential tool for fostering thinking skills and reinforcing intrinsic motivation among medical students. Hence future curricula need to have some room for self-driven learning which has outstanding results because students then actively participate in the learning process and can see themselves achieving personal goals. On the whole, a predominantly structured program with some segments of self-driven learning would presumably be an optimal option for an adequate learning experience in the operating room.

A second critical component, for the course and lesson design which emerged through Delphi rounds, is choosing the ‘right’ model for OR-based learning. Many models are currently being used for student and resident learning in the OR, which varies based on agency – the freedom for the student to decide learning objectives and modalities of learning. One commonly used is Robert’s Briefing-Intraoperative Teaching-Debriefing (BID) model [1]. This model provides diversity and opportunities for students to drive the learning process. Upon reviewing our study’s results, it was clear that Robert’s Briefing-Intraoperative Teaching-Debriefing model for teaching at the resident and student levels, was a popular choice among students. This technique is quite useful for imparting learning about surgical procedures—many of the participants of our study concorded with this model’s utility. This model defines the benefits of standardization of medical graduates. The 4C/ID model’s utility can also be employed successfully for learning skills within the simulated or standardized environment. However, this model’s application or the apprenticeship model for students may not be practically feasible considering the number of students, safety and ethical issues, and time constraints. Hence for every learning encounter or designed activity, an appropriate model would require selection, and the encounter’s structure would change accordingly.

The content selection should be shared between the teacher and the student and should focus on cognitive, psychomotor, and affective learning domains. The content and learning should be synchronized with simulation-based lab work, virtual patients, and standardized patient activities. The learning objectives should be clear, practical, and achievable within the OR learning environment framework and communicated timely. As described commonly in medical education, they should be *s*pecific, *m*easurable, *a*chievable, *r*elevant, *t*argeted, and *t*ime-bound (SMARTT). The initial orientation session should focus on all relevant aspects of the operating theater, its staff, OR protocols & its working. It should provide a detailed framework of the whole rotation, methodologies of teachings being used, modes of assessment, and interaction/synchronization with the skill lab work.

The role of teachers within the OR setting is of paramount importance. A surgeon’s interest, attitude, competence, and enthusiasm in turn enhance students’ understanding and performance which positively influences OR-based learning.

**Fig. 3:**
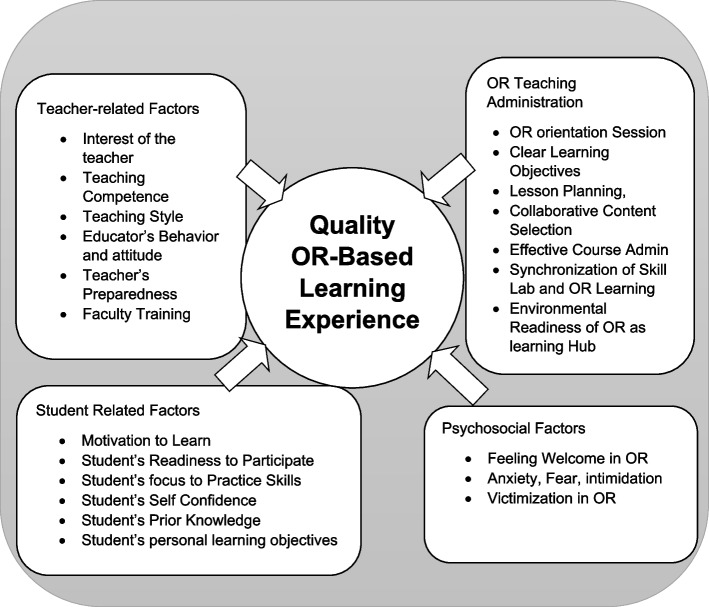
Schematic Model of Interplay of Stakeholders & Factors Affecting Quality of Operating Room Based Learning. Primary responsibility for any educational experience in OR lies on administering teacher, who is involved in student-centered content selection, lesson planning, and administration, which hinges on quality of trained faculty and administrative support. Balance of quality of OR-based learning experience is achieved by collective interplay of student & teacher-related factors and psychosocial factors, where teacher’s interest & competence and student’s motivation and desire to learn play central role

Table 3 gives an account of consensus guidelines for curricular design and lesson design within the operating room environment. Figure 3 shows a schematic diagram where the interplay of the factors and the stakeholders has been graphically depicted.

In summation, this comprehensive set of guidelines provides a focused and deliberate approach to the OR learning. While it can be a difficult task to implement these guidelines, these steps are doable and will eventually steer towards a more positive and favorable prospect for our future medical students. The introduction of students to clear, practical and achievable learning objectives and an orientation session prior to their theatre placement, instructing them to prepare topics beforehand and the interactive sessions between the students & the teacher according to Robert’s BID Model are some of the effective steps that can be employed. Moreover, the interest and dedication of the students & the teacher, the use of technology and simulation labs, and the constructive feedback after each interactive session are the building blocks of a standardized the OR learning environment.

### Limitations

Although there is a reasonable amount of literature available related to the factors that influence medical students learning, the data is contextual. The data is based on opinions without sound scientific evidence and may not be generalizable. Some studies have a low response rate signifying non-response bias that limits both the studies’ reliability and validity.

This study is also based on the participants’ perceptions and thoughts. It possesses inherent flaws of the Delphi technique, which is again opinion-based data collection that cannot completely exclude personal biases. In the future, it would be necessary to explore various models of learning and teaching within the OR setting.

## Conclusion

Operating room-based learning is complex and multifactorial and historically has remained challenging for both teachers and students. It is influenced by a number of factors. Structured learning through optimized course/lesson planning, content selection, assessment, and administration are decisive in determining the quality of OR learning experience. The teacher**’**s interest, attitude, and students**’** desire and preparedness to learn, play a central role in OR-based student learning, suggesting an enhanced need for adequate faculty training. Similarly, emotional, socio-environmental, and organizational factors can influence students**’** learning in a significant way. Based on these factors and the stakeholders**’** interplay, a new model for undergraduate student learning in OR has been proposed. In this model, the teacher**’**s role is responsible despite being student-centered, and guidelines for the OR curricular designs have been developed. A structured learning process within the OR setting is mandatory. Optimized lesson planning, content selection, assessment, and administration needs improved faculty training to produce standardized medical graduates. Guidelines have been developed to address essential components of quality student learning in OR and design structured clinical encounters within the OR for a more meaningful and enriched learning experience.

## Supplementary Information

**Additional file 1: Appendix 1.** (Questionnaire for Students).

**Additional file 2: Appendix 2.** (Questionnaire for Experts).

**Additional file 3: Appendix 3.** Operating Room (OR) Based Learning: Prioritizing the Factors Affecting Student Learning Experience.

**Additional file 4: Appendix 4.** Operating Room (OR) Based Learning: Exploring the Factors Affecting Student Learning Experience.

**Additional file 5: Appendix 5.** Table: Factors Affecting Student Learning in OR environment—Qualitative Analysis of the Delphi Data.

## Data Availability

Data supporting the findings of this study are available from the corresponding author (Talat Waseem) on request at (talat.waseem@sihs.org.pk; twaseem@gmail.com).

## Abbreviations

OR: Operating Room
I-CVI: Item-level Content Validity Index
BID model: Briefing, Intraoperative teaching and Debriefing model
STEP model: Peyton’s 4 Step Model
4C/ID Model: **“**4C**”**-four components, **“**ID**”**-Instructional Design
