# Supplementary material for: Enriching operating room based student learning experience: exploration of factors and development of curricular guidelines
Source: BMC Med Educ. 2022 Oct 26;22:739. doi: 10.1186/s12909-022-03793-x (PMC9597956; doi:10.1186/s12909-022-03793-x)
Supplement: Supplementary file 1 — Additional file 1: Appendix 1. (Questionnaire for Students). [file 12909_2022_3793_MOESM1_ESM.docx]

**Appendix I (Questionnaire for Students)**

**Operating Room (OR) Based Learning: Exploring Relative Value of Factors Affecting Student Learning Experience**

Dear Participant

Operation Theater is a dynamic, high-pressure setting where work-place based learning is challenging, all within an unfamiliar environment for students to acquire knowledge. Operation theater based student learning is influenced by number of factors which likely include emotional, socio-environmental, organizational factors and factors related to educational relevance and surgical educator. Identification and relative value of the factors influencing in OR based learning would be important to design structured clinical encounters within OR for further meaningful and enriched learning experiences.

We are conducting a study to understand the relative value of various factors that influence student learning in Operating Room (OR) based setting. The study would involve opinions from students, experts of the surgical field and the experts in medical education. These opinions would be debated in form of rounds to reach to a consensus (Delphi Study).

Please complete this questionnaire. Your opinions would be anonymously circulated among the panel members and your identity would be kept secret.

| Name |  | Age/ Sex |  |
| --- | --- | --- | --- |
| Institution |  | **Year** |  |
|  |  |  |  |
| Email |  | **Phone Number** |  |
|  |  |  |  |

**Questionnaire for Students**

| Themes | Sub themes | Questions |
| --- | --- | --- |
| Learning Objectives/ Educational Relevance | 1. Intra-operative Teaching Session Los 2. Clarity of Learning Objectives 3. Practicality of Learning Objectives 4. Synchronization of Learning Objectives with rest of Teaching 5. Subjective or Personal Learning Objectives | 1. How does the institute intimate you about the OR lesson learning objectives in advance? 2. How important it is for you to know about the OR lessons being taught in advance? 3. How does the institute provide you with relevant structured lessons prior to OR session? 4. Does your institute inform you about the operation list before operation and prepare you about the cases you are going to observe in the OR? 5. How does the institute arrange for the students to take history of the relevant cases prior to operation? 6. How satisfied you feel about the teaching during operation? 7. Does the surgeon ask you questions about the ongoing procedure to keep you involved in the process? 8. How does the surgeon keeps you involved in the procedure during operation? 9. Do you a have question answer session before and after surgical procedure to clear any confusion related to the procedure? 10. How does you institute collect your feedback about the session in OR? 11. Are you clear about what you are going to learn in the OR before the start of your rotation in OR? 12. Are you clearly informed about the learning objectives of OR lessons in writing? 13. Do you feel that you are not being taught what you were supposed to learn in OR? 14. Are you learning what you intended to learn as per your learning objectives provided? Please Explain. 15. Do you experience a gap between the planning of learning goals and their implementation in OR sessions? 16. Do your OR and class lessons address the same topic at the same time? If not, how it affect your OR learning? 17. Do you think it is practically possible to have OR and class lessons on the same topic simultaneously? Give the reasons. 18. Do you feel the need for your own personal learning objectives in OR sessions apart from the institutional learning objectives? If so, please justify. |
|  |  | 1. Out of the five described subthemes, how would you rate their importance on scale 1-10 on Likert scale 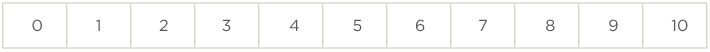 2. Intra-operative Teaching Session Los 3. Clarity of Learning Objectives 4. Practicality of Learning Objectives 5. Synchronization of Learning Objectives with rest of Teaching 6. Subjective or Personal Learning Objectives  - If you think of any other factor related to educational relevance or learning objectives, please describe it in detail. |
| Educator Related Factors | 1. Interest of Educator 2. Behavior / Attitude 3. Teaching Competence / Experience 4. Teaching Style 5. Preparedness | 1. How does interest of the surgeon impact on your OR sessions? 2. Do you think being interactive the surgeon can facilitate learning in OR more effectively? Please explain. 3. How do you feel when the surgeon is so busy in surgery and is unable to explain it to you fully? 4. How does the behavior of the teachers impacts your OR learning? 5. Does being cordial by surgeon facilitate your interest and learning in OR? 6. Does the surgeon encourage active participation or demands silence observation by you? How does it impact your learning? 7. How does being authoritative or collaborative of the surgeon in OR affects your learning? 8. How does the experience of the surgeon impact on your OR learning? 9. How does the competence of the surgeon affect you OR learning? 10. What teaching methods does your teacher use in OR learning? Are they helpful? 11. How does the teaching style of surgeon affect your learning in OR? 12. How does the preparedness of teacher impact your learning in OR sessions? 13. Do you think the OR sessions are more beneficial when your teacher has prepared for it before hand? |
|  |  | 1. . If you think of any other factor related to educator, please describe it in detail. 2. Out of the five described subthemes, how would you rate their importance on scale 1-10 on Likert scale 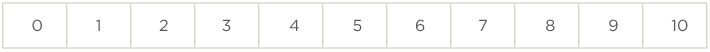  - Interest of Educator - Behavior / Attitude - Teaching Competence / Experience - Teaching Style - Preparedness |
| Organizational Factors | 1. OR Dynamics Orientation Session 2. Readiness of OR as Learning Hub 3. Synchronization with Simulation Lab 4. Visualization | 1. Does your teacher take you along for an introductory session about the OR lessons at start of your Or rotation? How helpful it is? 2. What was the content of the introductory lecture you attended at the start of your OR training? 3. How did your teacher teach you about guidelines, protocols and ethics you need to follow during OR sessions? 4. What provisions you think are important for the learning in OR? 5. How does your institute manage OR lesson for students? 6. Is there any separate class room within Operation Theater to teach you? If so how is it helpful? If not is there a need for it? Please explain. 7. Is there a skill lab available for learning skills in OR? How does it make difference? 8. How does the institute ensure that what you learn in skill lab is also taught in OR? 9. How is this synchronization helpful for your learning? 10. How clearly you can see the surgical procedure in OR? 11. If yes, how is it being facilitated at your institute, if not what are the hurdles? |
|  |  | 1. If you think of any other factor related to educator, please describe it in detail. 2. Out of the four described subthemes, how would you rate their importance on scale 1-10 on Likert scale 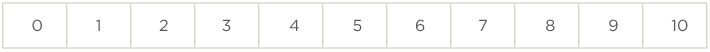 3. OR Dynamics orientation session 4. Readiness of OR as a learning hub 5. Synchronization with simulation lab 6. Visualization |
| Emotional Factors | 1. Anxiety 2. Intimidation or Fear 3. Feeling Welcome 4. Feeling Burdensome 5. Victimization 6. Confidence 7. Motivation | 1. How do you feel emotionally in OR? 2. When do you feel stressed in OR and how do you deal with it? 3. How is your energy level during OR session? 4. How your energy level affect your performance in OR? 5. What intimidates you in OR? 6. When do you feel awkward or self conscious in OR and how do you cope with it? 7. Do you feel being welcome in OR? How does it impact your participation and learning in OR? 8. In which situations, you feel burdensome and feel like disturbing the surgical process and how do you manage it? 9. Do you feel victimized in OR sessions? Please explain. 10. How actively you participate in OR sessions? 11. Are you reluctant to ask questions if something is not clear? 12. How does the teacher boost you confidence level in OR sessions? 13. What motivates you to attend OR sessions regularly? 14. What prevents you from attending OR sessions? |
|  |  | 1. If you think of any other factor related to educator, please describe it in detail. 2. Out of the seven described subthemes, how would you rate their importance on scale 1-10 on Likert scale 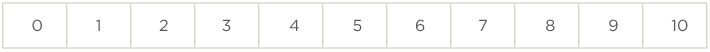 3. Anxiety 4. Intimidation or Fear 5. Feeling Welcome 6. Feeling Burdensome 7. Victimization 8. Confidence 9. Motivation |
|  |  |  |
|  |  |  |

|  | Factors Affecting Student OR Learning | Rate on a Likert Scale of 0-10 on the relative importance of various factors | What do you suggest to tackle this issue |
| --- | --- | --- | --- |
| 1  2  3  4  5  6  7  8  9  10  11  12  13  14  15  16  17  18  19  20  21  22  23 | Intra-operative Teaching Session Learning Objectives  Clarity of Learning Objectives  Practicality of Learning Objectives  Synchronization of Learning Objectives with rest of Teaching  Subjective or Personal Learning Objectives  Interest of Educator  Teacher’s Behavior / Attitude  Teaching Competence / Experience  Teaching Style  Teacher Preparedness  OR Dynamics Orientation Session  Readiness of OR as Learning Hub  Synchronization with Simulation Lab  Visualization  Anxiety  Intimidation or Fear  Feeling Welcome  Feeling Burdensome  Victimization  Confidence  Motivation | 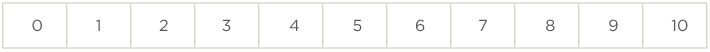  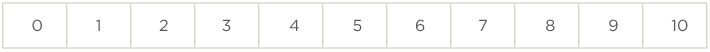  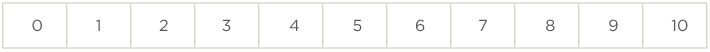  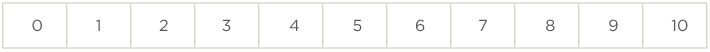  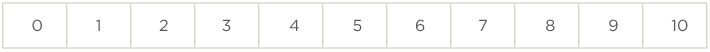  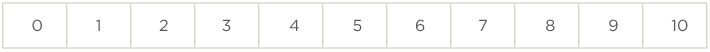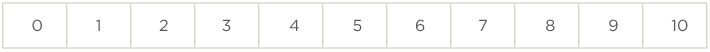  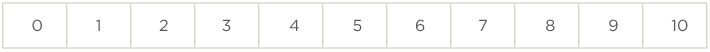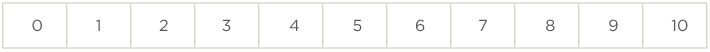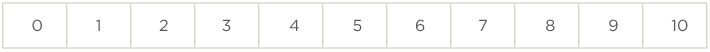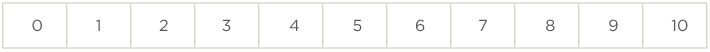  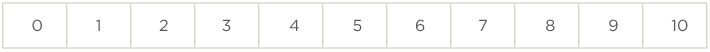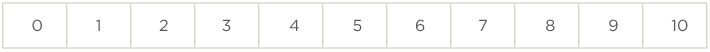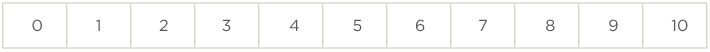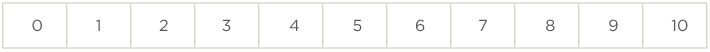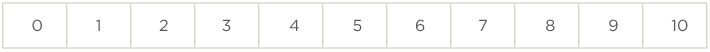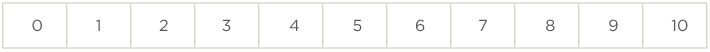 |  |
