# Supplementary material for: Enriching operating room based student learning experience: exploration of factors and development of curricular guidelines
Source: BMC Med Educ. 2022 Oct 26;22:739. doi: 10.1186/s12909-022-03793-x (PMC9597956; doi:10.1186/s12909-022-03793-x)
Supplement: Supplementary file 2 — Additional file 2: Appendix 2. (Questionnaire for Experts). [file 12909_2022_3793_MOESM2_ESM.docx]

**Appendix II (Questionnaire for Experts)**

**Operating Room (OR) Based Learning: Exploring Relative Value of Factors Affecting Student Learning Experience**

Dear Participant

Operation Theater is a dynamic, high-pressure setting where work-place based learning is challenging, all within an unfamiliar environment for students to acquire knowledge. Operation theater based student learning is influenced by number of factors which likely include emotional, socio-environmental, organizational factors and factors related to educational relevance and surgical educator. Identification of the factors influencing in OR based learning would be important to design structured clinical encounters within OR for further meaningful and enriched learning experiences.

We are conducting a study to understand the relative value of various factors that influence student learning in Operating Room (OR) based setting. The study would involve opinions from students, experts of the surgical field and the experts in medical education. These opinions would be debated in form of rounds to reach to a consensus (Delphi Study).

Please complete this questionnaire. Your opinions would be anonymously circulated among the panel members and your identity would be kept secret.

| Name |  | Age/ Sex |  |
| --- | --- | --- | --- |
| Institution |  | **Designation** |  |
| Experience in Years (Surgery and Allied) |  | **Experience in Medical Education in Years in (Surgery & Allied)** |  |
| Email |  | **Phone Number** |  |
|  |  |  |  |

| Themes | Sub themes | Questions |
| --- | --- | --- |
| Learning Objectives/ Educational Relevance | 1. Intra-operative Teaching Session Los 2. Clarity of Learning Objectives 3. Practicality of Learning Objectives 4. Synchronization of Learning Objectives with rest of Teaching 5. Subjective or Personal Learning Objectives | 1. How does the institute intimate student about the OR lesson learning objectives in advance? 2. How important it is for students to know about the OR lessons being taught in advance? 3. How does the institute provide student with relevant structured lessons prior to OR session? 4. Does your institute inform student about the operation list before operation and prepare them about the cases you are going to observe in the OR? 5. How does the institute arrange for the students to take history of the relevant cases prior to operation? 6. How satisfied you feel about the teaching during operation? 7. How do you keep your student involved in the procedure during operation? 8. Do you ask questions about the ongoing procedure to keep students involved in the process? 9. Do you a have question answer session before and after surgical procedure to clear any student confusion related to the procedure? 10. How does you institute collect student feedback about the session in OR? 11. Are you clear about what you are going to teach in the OR before the start of student rotation in OR? 12. Do you clearly inform students about the learning objectives of OR lessons in writing? Please explain how? 13. Do you feel that you are not teaching what you were supposed to teach in OR? 14. Are your students learning what you intended to teach as per your learning objectives provided? Please Explain. 15. Do you experience a gap between the planning of learning goals and their implementation in OR sessions? 16. Do your OR and class lessons address the same topic at the same time? If not, how it affect your OR learning? 17. Do you think it is practically possible to have OR and class lessons on the same topic simultaneously? Give the reasons. 18. Do you feel that students need their own personal learning objectives in OR sessions apart from the institutional learning objectives? If so, please justify. |
|  |  | 1. Out of the five described subthemes, how would you rate their importance on scale 1-10 on Likert scale 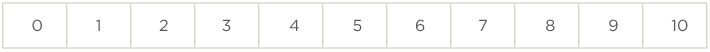 2. Intra-operative Teaching Session Los 3. Clarity of Learning Objectives 4. Practicality of Learning Objectives 5. Synchronization of Learning Objectives with rest of Teaching 6. Subjective or Personal Learning Objectives  - If you think of any other factor related to educational relevance or learning objectives, please describe it in detail. |
| Educator Related Factors | 1. Interest of Educator 2. Behavior / Attitude 3. Teaching Competence / Experience 4. Teaching Style 5. Preparedness | 1. How does interest of the surgeon impact on student OR sessions? 2. Do you think being interactive the surgeon can facilitate learning in OR more effectively? Please explain. 3. How do you feel when you as a surgeon are so busy in surgery and are unable to explain it to your students fully? 4. How does the behavior of the teachers impacts student OR learning? 5. Does being cordial by surgeon facilitate student interest and learning in OR? 6. Do you encourage active participation or demand silence observation by students? How does it impact student learning? 7. How does being authoritative or collaborative of the surgeon in OR affects student learning? 8. How does the experience of the surgeon impact on your OR learning? 9. How does the competence of the surgeon affect you OR learning? 10. What teaching methods do you use in OR learning? How they are helpful? 11. How does the teaching style of surgeon affect your learning in OR? 12. How does the preparedness of a teacher impact student learning in OR sessions? 13. Do you think the OR sessions are more beneficial when you have prepared for it before hand? |
|  |  | 1. . If you think of any other factor related to educator, please describe it in detail. 2. Out of the five described subthemes, how would you rate their importance on scale 1-10 on Likert scale 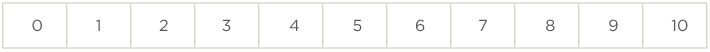  - Interest of Educator - Behavior / Attitude - Teaching Competence / Experience - Teaching Style - Preparedness |
| Organizational Factors | 1. OR Dynamics Orientation Session 2. Readiness of OR as Learning Hub 3. Synchronization with Simulation Lab 4. Visualization | 1. Do you take your students along for an introductory session about the OR lessons at start of student OR rotation? How helpful it is? 2. What was the content of the introductory lecture you delivered at the start of your OR training? 3. How do you teach your students about guidelines, protocols and ethics you need to follow during OR sessions? 4. What provisions you think are important for the learning in OR? 5. How does your institute manage OR lesson for students? 6. Is there any separate class room within Operation Theater to teach you? If so how is it helpful? If not is there a need for it? Please explain. 7. Is there a skill lab available for learning skills in OR? How does it make difference? 8. How does the institute ensure that what student learns in skill lab is also taught in OR? 9. How is this synchronization helpful for student learning? 10. How clearly your students can see the surgical procedure in OR? 11. If yes, how is it being facilitated at your institute, if not what are the hurdles? |
|  |  | 1. If you think of any other factor related to educator, please describe it in detail. 2. Out of the four described subthemes, how would you rate their importance on scale 1-10 on Likert scale 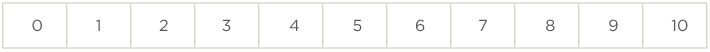 3. OR Dynamics orientation session 4. Readiness of OR as a learning hub 5. Synchronization with simulation lab 6. Visualization |
| Emotional Factors | 1. Anxiety 2. Intimidation or Fear 3. Feeling Welcome 4. Feeling Burdensome 5. Victimization 6. Confidence 7. Motivation | 1. How do your students feel emotionally in OR? 2. When do your students feel stressed in OR and how do you deal with it? 3. How is your students’ energy level during OR session 4. How your energy level affect your performance in OR? 5. What intimidates your students in OR? 6. When do your students feel awkward or self conscious in OR and how do you cope with it? 7. Do your students feel being welcome in OR? How does it impact their participation and learning in OR? 8. In which situations, your student feels burdensome and feel like disturbing the surgical process and how do you address it? 9. Do your students feel victimized in OR sessions? Please explain. 10. How actively your students participate in OR sessions? 11. Are your students reluctant to ask questions if something is not clear? 12. How do you boost student confidence level in OR sessions? 13. What motivates your students to attend OR sessions regularly? 14. What prevents your students from attending OR sessions? |
|  |  | 1. If you think of any other factor related to educator, please describe it in detail. 2. Out of the seven described subthemes, how would you rate their importance on scale 1-10 on Likert scale 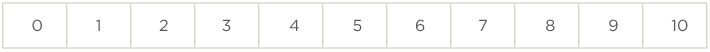 3. Anxiety 4. Intimidation or Fear 5. Feeling Welcome 6. Feeling Burdensome 7. Victimization 8. Confidence 9. Motivation |
|  |  |  |
|  |  |  |

|  | Factors Affecting Student OR Learning | Rate on a Likert Scale of 0-10 on the relative importance of various factors | What do you suggest to tackle this issue |
| --- | --- | --- | --- |
| 1  2  3  4  5  6  7  8  9  10  11  12  13  14  15  16  17  18  19  20  21  22  23 | Intra-operative Teaching Session Learning Objectives  Clarity of Learning Objectives  Practicality of Learning Objectives  Synchronization of Learning Objectives with rest of Teaching  Subjective or Personal Learning Objectives  Interest of Educator  Teacher’s Behavior / Attitude  Teaching Competence / Experience  Teaching Style  Teacher Preparedness  OR Dynamics Orientation Session  Readiness of OR as Learning Hub  Synchronization with Simulation Lab  Visualization  Anxiety  Intimidation or Fear  Feeling Welcome  Feeling Burdensome  Victimization  Confidence  Motivation | 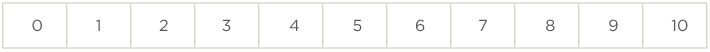  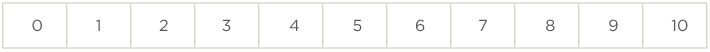  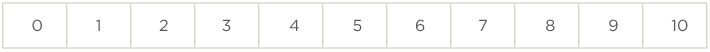  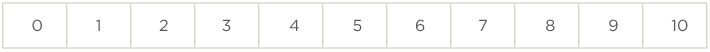  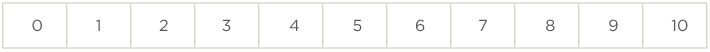  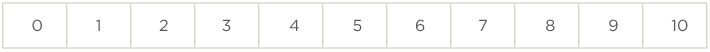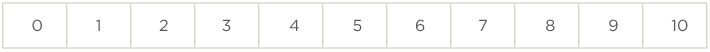  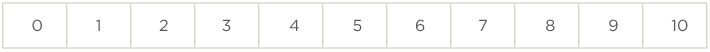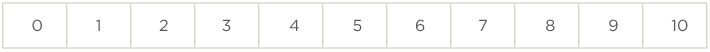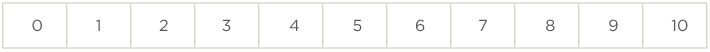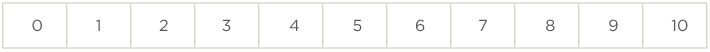  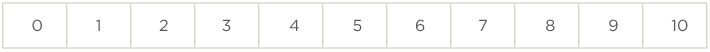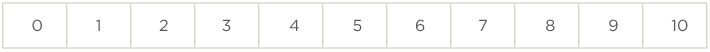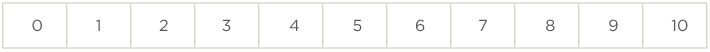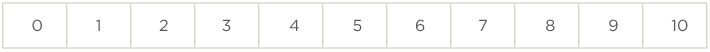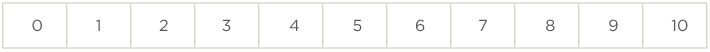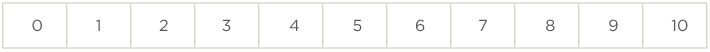 |  |
