# Supplementary material for: Enriching operating room based student learning experience: exploration of factors and development of curricular guidelines
Source: BMC Med Educ. 2022 Oct 26;22:739. doi: 10.1186/s12909-022-03793-x (PMC9597956; doi:10.1186/s12909-022-03793-x)
Supplement: Supplementary file 3 — Additional file 3: Appendix 3. Operating Room (OR) Based Learning: Prioritizing the Factors Affecting Student Learning Experience. [file 12909_2022_3793_MOESM3_ESM.docx]

**Operating Room (OR) Based Learning: Prioritizing the Factors Affecting Student Learning Experience**

**What are we doing in this study?** To know a brief summary of the research design of this study, go to **Research Protocol** attached along with this document.

**Quantitative & Qualitative Analysis of the Delphi Questionnaire: Delphi Round 1 Report**

14 Students and 15 experts with experience in surgical / medical education participated in this first round of the study. Quantitative and qualitative analysis was done based on the filled Delphi questionnaire and following observations were made. Most of the factors described based on the literature review were considered important. Most of the factors have been rated as either ‘Quite Important’ or ‘Highly Important’. Only one factor related to student driven personal learning objectives is considered ‘Somewhat Important’. The relative rating is given in the table (Means+/-SD).

Qualitative responses were analyzed by thematic analysis given in the table below. Highlighted items are subthemes which emerged through this thematic analysis. At the end of this table, two new emerging themes along with subthemes in form of ‘Lesson Planning & Administration’ and ‘Student Related Factors’ have been added.

| Themes | Subthemes | Relative Importance on Likert Scale (1-10)  Mean+/-SD | % of Participants who rate factors either Quite or Highly Important | Qualitative Analysis: Final Codes |
| --- | --- | --- | --- | --- |
| Learning Objectives | 1. Communication of Learning Objectives for OR learning | 9.138 ±.8752 | 99.9% | 1. It is important to provide guideline to the students about OR learning. 2. Students gain insight about what they are going to learn in OR 3. It helps in setting and achieving learning objectives. 4. It rules out the distracting factors 5. It facilitates OR[-based] learning |
|  | 2. Clarity of Learning Objectives | 9.103± .9763 | 100% | 1. It provides better understanding about OR based learning. 2. It helps students to learn new things and relate to basic clinical knowledge. 3. It minimizes the anxiety and confusion among students 4. It increases student’s participation and vigilance in OR. 5. It makes students goal oriented, focused and they can set an achieve their goals. 6. It enables them to prepare for exam and focus the important topics. 7. It improves quality of learning and teaching. 8. It improves student’s confidence and clinical skills 9. Learning objectives should be fewer so they can be achievable. 10. Clarity of learning is nor possible in clinical cases as some times they overlap. 11. The weightage of learning objectives facilitates OR based learning |
|  | 3. Feasibility of learning objectives to be realistically achievable | 8.621± 1.3993 | 89.6% | 1. Learning objectives should be simple and designed according to the learning abilities of an average student, local facilities and logistics 2. If the learning objectives are feasible and achievable, the students would show lack of interest, depression and anxiety. 3. The achievements of the learning objectives result in effective learning. 4. When the learning objectives are not feasible, their implementation would be difficult. 5. Feasible and realistically achievable learning objectives motivate students. 6. Learning objectives provide student a guideline about OR based learning by emphasizing the important aspects of the training. |
|  | 4. Synchronization of the learning objectives with rest of the teaching. | 8.414± 1.7012 | 86.1% | 1. It facilitates OR based learning. 2. It guides and prepares students for OR training 3. It results in easy and better understating of the concepts 4. Synchronization improves quality of teaching 5. Synchronization helps students to filter unnecessary information and focus on the important aspects 6. Synchronization enhances understanding 7. It saves time. 8. It motivates students to participate in OR session. |
|  | 5. Importance of Personal Learning Objectives in OR Learning | 6.621± 1.5449 | 65.5% | 1. Personal Learning objectives encourages self-directed learning among students. 2. It shows student’s motivation, preparedness and help them in self-assessment. 3. Personal learning objectives should be realistic 4. Personal learning objectives enable students to improve their weaknesses 5. It should be synchronized to the course objectives to facilitate student learning. 6. Personal learning objectives are important for residents but not for the undergraduate students. 7. Personal learning objectives slow down learning when they are not synchronized to the course objectives. The students will only strive for their personal learning objectives. |
| Educator Related Factors | 1. Interest of educator | 9.414± .7328 | 100% | 1. Interest of educator motivates students 2. Educator’s interest keeps students involved and interested 3. Interest of educator shows his determination and UpToDate knowledge 4. Interest of educator facilitates student learning 5. An Or session without interest of educator is a waste of time 6. The student will not achieve their learning objectives 7. The aggressive behavior of educator effects negatively on OR based learning 8. The positive attitude of the educator inspires students to chose specialty after their ideal. |
|  | 1. Importance of educator’s behavior and attitude | 9.069± 1.0667 | 96.5% | 1. Educator’s behavior and attitude facilitates learning in OR 2. The aggressive attitude or behavior of the educator results in lack of interest by the student and missing [their] OR sessions. 3. Educator should provide assistance even after OR session to answer questions for better understanding of the concepts 4. Educator should have positive attitude and behavior as the students consider them their role models. 5. Students feel intimidated by aggressive behavior of the educator. It affects their class participation negatively. The students will not ask questions. It will frustrate them. 6. Educator’s behavior provides positive learning environment. 7. Educator needs to be helpful, should show empathy, should be cooperative and non-judgmental. |
|  | 1. Competency of educator | 9.000± 1.1952 | 93% | 1. Teacher needs to be experienced. It will help students to achieve their learning objectives, facilitate learning 2. The teacher should demonstrate the skills in easy and professional way. 3. It motivates the students, sparks interest. The [competent] teacher transfers information effectively. 4. The old teachers’ teaching style is outdated, not according to the mental abilities of their students 5. You learn a lot from an incompetent teacher by assuring what not to do 6. Competency of the educator improves teacher’s confidence. 7. Competency is a teaching attitude and is more important than experience. 8. Experience of the teacher is useless if he cannot teach good. |
|  | 1. Importance of teaching style | 8.793± 1.1765 | 96.5% | 1. Good teaching style enhances students learning, increased participation and attendance. 2. New teaching strategies improve student’s interest level 3. Teaching style should be friendly, effective and versatile 4. Teacher should know students learning style too to teach according to students’ mental abilities. Teaching according to his [teacher’s] level will not be beneficial for the students. 5. Teacher should not cover lots of topics in one session. 6. Ongoing commentary during procedure will boost students’ interest and attendance |
|  | 1. Importance of teacher’s preparedness | 8.379± 1.8403 | 89.5% | 1. Preparedness of teacher enhances OR based student learning. 2. It improves quality of teaching 3. If teacher is not prepared, it would demotivate the students and they cannot achieve their learning objectives 4. The preparedness of the teachers shows his dedication and helpful attitude. 5. It is important to clear to student confusions and keep them involved in OR sessions. 6. If the teacher will not prepare, he would miss important aspects of the topic. |
|  | 1. Content Selection can have significant impact on student’s | 8.931± 1.1628 | 93% | 1. The content should be selected according to the learning requirement of the students. 2. The content should be dynamic, exam oriented, practical, custom-made, not imported from other curricula. 3. Frequently required skills in OR should be emphasized. 4. The content should evolve over the time with the feedback provided by the students and the teachers and to synchronize teaching and learning. 5. Repetition of the content will result in lack of interest. 6. Exam oriented lectures would be useful, 7. There should be follow up classes next day of the procedure to enhance students learning 8. Pre and postoperative care should be focused 9. In OR the students should be tested on their theoretical and practical knowledge |
| Organizational Factors | 1. Significance of OR orientation session. | 8.862± 1.2740 | 93% | 1. It provides a guideline about OR protocol and helps students to familiarize with OR. 2. It develops students’ interest and involvement in OR training. 3. It saves time 4. It communicates what is expected from the students 5. It helps the students to set their learning objectives. 6. Orientation session is not important, students should figure out everything adventurously. 7. If a student doesn’t follow the protocols after orientation session, they can be held responsible. 8. Session on OR etiquettes would help students |
|  | 1. Importance of environmental readiness | 8.414± 1.4272 | 93.2% | 1. It improves quality of learning 2. Or should be equipped with all necessary resources such as LCDs and speakers so the students can observe the procedure properly. 3. Without environmental readiness of the OR, students would be distracted and not involved. 4. Environmental readiness is not required and it will not affect OR based learning. 5. It is practically impossible because theater is a busy place. |
|  | 1. Synchronization simulation / Lab activities with OR lessons | 8.586± 1.1501 | 93.1% | 1. It improves OR learning by reinforcing the concepts 2. It will help the students to identify their strengths and weaknesses. 3. It will develop student’s interest and polish their skills 4. It will boost the students’ confidence. 5. It cannot replace the real situation; student will fail practically and they will feel intimidated because of lack of practice [in real environment]. |
|  | 1. Importance of adequate visualization in student learning | 8.897± 1.3187 | 93.2% | 1. Better visualization in OR will result in better understanding of the concepts. 2. Equipment would improve visualization in OR such as LCDs etc. 3D visualization is important for OR based learning. 3. The large number of students make it difficult to visualize the procedure and students lose their interest. 4. If some students miss the procedure one time, they can be shown the video of the same procedure or they can visualize this next time as the clinical cases repeat frequently in OR. |
| Psychosocial factors | 1. Impact of anxiety in OR environment | 7.897± 1.4963 | 82.7% | 1. Anxiety slows down students learning and distracts students and teachers. 2. The teacher can help students to manage their anxiety. 3. The aggressive behavior of the surgeon indices anxiety in OR 4. The students are not anxious but they are excited about OR learning 5. Wearing scrubs boosts confidence and anxiety subsides. 6. Preparing the students before OR learning will minimize their anxiety. 7. The bust theater environment induces anxiety 8. Dealing with human life is stress inducing 9. It results in low attendance and students will not feel welcomes 10. The students need time to adjust to OR environment. |
|  | 1. Effect of fear and intimidation in OR learning environment | 8.483± 1.6609 | 86.2% | 1. Fear hampers learning while positive OR environment facilitates learning. 2. Fear of being criticized is common among students 3. Fear results in missing sessions, low participation, lack of interest, poor self-esteem and demotivation. 4. Fear can be managed by encouragement and training 5. The aggressive behavior of the student indices fears as some surgeons use this as teaching strategy but they should be banned instead. 6. Fear leads to psychological burnout as residents are already exhausted after 36 hours of duty. 7. The orientation session reduces fear among students |
|  | 1. Impact of feeling welcome in OR | 9.034± 1.0171 | 96.4% | 1. Welcoming students in OR will increase attendance, participation, interest and make them feel part of the team 2. Students are insulted for not answering the questions bullied and harassed by surgeons 3. Students need to be tuned to calm down. 4. Students should be welcomed in OR by abiding all the rules and regulations of OR. It is very important the rules in OR. 5. Teachers use aggression as teaching strategy which should not be practiced. 6. The surgeons should perceive their students as future doctors and should train them well. 7. The teacher s should be mentally relaxed |
|  | 1. Impact of victimization in OR environment | 8.828± 1.2837 | 93.2% | 1. Victimization of the students will result in, low attendance, demotivation, low participation, fear, loss of interest and excitement 2. Staff should not be allowed to that. It is teacher’s weakness being not able to control the environment. 3. It affects student’s efficacy 4. The student feels lost and drained 5. Sometimes it works positively as the students work cautiously and carefully. |
|  | 1. Impact of self confidence | 8.690± 1.2846 | 93.1% | 1. It is a positive factor that improves quality of learning 2. Overconfidence affects negatively 3. Confident students learn better, show good social skills and participate more. 4. Nonjudgmental teaching style improves student’s confidence 5. Confident students work hard |
|  | 1. Impact of student motivation | 8.897± 1.3455 | 93% | 1. It facilitates OR learning 2. Interest is more important that the motivation. 3. Positive reinforcement increases motivation 4. It increases student attendance, helps them to achieve their learning objectives 5. Self-motivation is more important than the external motivation 6. The teachers are responsible to motivate their students if they cannot they are a failure 7. Motivation is required to achieve competency. |
|  |  |  |  | **NEW EMERGINING THEMES & SUBTHEMES** |
| Lesson Planning & Administration | 1. Class Size & Equal Participation |  |  | 1. Number of students should be less 2. All students should get equal opportunity to participate. |
| Student Related Factors | 1. Student’s Prior Knowledge 2. Student’s Pre-lesson preparation 3. Student’s Readiness to participate 4. Student’s Focus on Practice |  |  | 1. Student’s prior knowledge and skills can be really beneficial in OR training. 2. The students [should] read topics before coming to OR sessions 3. Students should watch videos before OR procedure[s] 4. Students should sleep well and [should be] medically fit 5. The student s should [exhibit] readiness to participate and assist 6. Self-regulation is [the] most required factor or quality 7. The students should practice more [to gain better skills] |
