# Supplementary material for: Enriching operating room based student learning experience: exploration of factors and development of curricular guidelines
Source: BMC Med Educ. 2022 Oct 26;22:739. doi: 10.1186/s12909-022-03793-x (PMC9597956; doi:10.1186/s12909-022-03793-x)
Supplement: Supplementary file 5 — Additional file 5: Appendix 5. Table: Factors Affecting Student Learning in OR environment—Qualitative Analysis of the Delphi Data. [file 12909_2022_3793_MOESM5_ESM.docx]

**Table: Factors Affecting Student Learning in OR environment—Qualitative Analysis of the Delphi Data**

| Themes (5) | Subthemes (27) | Qualitative Analysis: Final Codes (79) | Representative Qualitative Statements |
| --- | --- | --- | --- |
| Course /Lesson Planning, Content Selection, Assessment & Administration | 1. OR learning should be structured *not opportunistic* | 1. Structured learning provides an opportunity for a uniform, standardized learning process 2. Opportunistic learning leads to non-uniform learning and possibly may exclude important learning objectives of the medical graduate | 1. *There are few important and essential learning objectives of a standardized medical graduate, which must be taught, otherwise you may end up producing unsafe doctors.* 2. *At least at the graduate level, learning should be standardized and faculty-driven so that all attributes of a safe doctor can be inculcated.* 3. *Opportunistic learning leads to non-uniform learning, thus missing important learning themes essential for a safe doctor.* 4. *The goals and objectives should be carefully selected; however, in the process of their delivery, student's input should be incorporated.* |
|  | 1. Content Selection should encompass essential aspects of graduate learning and should be faculty-driven with student input | 1. The content should be selected according to the learning requirement of the students. 2. The content should be dynamic, exam oriented, practical, custom-made, not imported from other curricula. 3. Frequently required skills in OR should be emphasized. 4. The content should evolve over the time with the feedback provided by the students and the teachers and to synchronize the rest of teaching and learning. 5. The content should be based on operative interventions with more live component 6. Teachers are more interested in teaching clinical decision making about various cases while students wish to be part of hands-on aspects 7. Repetition of the content will result in a lack of interest. 8. The assessments should be aligned with the content and should assess psychomotor and affective component. | 1. *Content selection is very important, and both teacher and the student should play a role in it.* 2. *The content selected for OR teaching is very important. OR learning is not by lecturing, tutoring, or performing SGDs. It is something very practical and obtaining the results on that specific moment. The content should be practically doable and should be according to the level of students involved.* 3. *Extremely important to filter the content to the student's level of understanding and keep the content exam-oriented. The content should be selected by a group presided by the teacher and students who have actually studied the subject and passed the exam as they are in a better position to highlight the important points and shortcomings.* 4. *It should be made by teachers after discussing with students. It should not be something fixed. it should be dynamic and should evolve with the feedback of students and teachers. The content should always be custom-made by the college/university itself, not to be imported from any other curriculum* 5. *I think, we can decide outcomes beforehand and then discuss it with the students, how would they like to achieve it? The student, however, should focus more on the clinical decision making and steps of management rather than the hands-on component.* 6. *It is highly important that a student is taught valid and the most pertinent content that is going to help him the most during his learning. Selecting appropriate content lies both with the teacher and the student. It is a result of intercommunication between them both to extract valid points regarding the content of the material that they should be taught.* 7. *Subject experts of that area should select the content. Content selection, to achieve desired outcomes should be in consultation with the students. This is a common finding that Students (especially Residents) always take interest and feel motivated in those procedures, which they think are essential for their level of training. So, to bridge the gaps in their training and become confident in performing a procedure, at par with their learning outcome, they perform reasonably well. So, content selection may be different with different batch of students (residents), depending upon their needs and requirements.* 8. *The teacher should select the content only after the discussion with the students. It should be according to the interest and mental capacity of the students. The content should be synchronized with learning objectives* 9. *Selection of appropriate cases with exam-oriented and high-yield cases for students and combined teaching with different fields and preoperative and postoperative patient care should be focused upon.* 10. *Practical and live sessions like in OR have a greater impact on learning rather than book or internet study. Hands-on part is always exciting.* 11. *I guess student may not have an idea about the content. So, teachers have an important role in selecting it.* 12. *The assessments should be aligned with the content selected and taught.* 13. *All students at the end of the year have to give the board exam. Passing these exams is usually the motivation for most students. The learning objectives as well as the way of teaching must be tailored to the pattern of those exams. It is pointless if all year round the student learns in a different manner and when exams come around, they cannot reproduce what they learned or how the exam is asking them to answer. Adoption of new techniques and way of teaching is good in its own right; however, I feel they are worthless if it clashes with the system that is already been established and is old fashioned. System of learning should be improved from the base at junior level so when they reach the higher level, they are well versed in these techniques that they can understand it and not be intimidated by it.* |
|  | 1. Designing and Communicating Learning Objectives for OR learning should be mandatory | 1. Communication of learning objectives improves student's thought process, focus and learning | 1. *Objectives are defined paths of learning so that students should know them, so [that] they can focus [on] learning towards achieving those specific objectives.* 2. *Objectives drive learning and give direction.* 3. *Knowing about learning objectives beforehand gives an orientation about what are we going to be through and what can we expect to learn in OR.* 4. *Specific learning objectives direct learning activity to achieve maximum retention of knowledge.* 5. *Knowing what parts of the procedure are to be observed with what amount of vigilance is important and filters out the information which is too specialized for the student's current level. Clear learning objectives will help students focus on the take-home message.* 6. *A target needs to be set up and known to all involved to get to that target.* 7. *Journey without knowing the destination leads to being lost. Knowing what the aim of each session will direct the interest as well as the questioning during that session.* |
|  | 1. Clarity of Learning Objectives | 1. The learning objectives should be clear and well-aligned to content overall learning goals 2. The learning objectives should be *s*pecific, *m*easurable, *a*chievable, *r*elevant, *t*argeted and *t*ime-bound (SMART) to enhance OR learning process. | 1. *Learning objectives set the goal [of lesson].* 2. *As ambiguity leads to confusion and anxiety.* 3. *Clarity brings action* 4. *SMART (Specific, Measurable, Achievable, Relevant and Targeted/Time-bound) objectives will help students to be more focused.* 5. *Assuring that learning objectives are clearly defined has importance in a way that OR-based learning requires active participation and vigilance of students* 6. *Clarity like "He [or she-student] should be able to dissect Callot's Triangle in easy Laparoscopic Cholecystectomy in predefined time" will be far better than the routine objective of " should assist Laparoscopic Cholecystectomy".* 7. *Clear learning objectives will help students focus on exam-oriented study of cases and will help retain information relevant to their level of understanding.* 8. *It may not be possible to "clearly define" objectives in all cases. Surgical objectives also involve rehearsing previously learnt material, so there is an element of overlap.* |
|  | 1. Feasibility of learning objectives to be realistically achievable | 1. Learning objectives should be simple and designed according to the learning abilities of an average student, local facilities and logistics 2. Feasible and realistically achievable learning objectives motivate students. | 1. *It is very important that students understand the basis of why they are observing the procedure and what they should take out of it at the end and what to focus on* 2. *More feasibility means easier explanation that a student can understand* 3. *Objectives should be achievable and realistic to keep the interest and stay motivated. The gap between the start and endpoint must be kept at an achievable level as to not fail in reaching it.* 4. *Being teachers, we are well aware of feasible learning objectives in our setup. We are well aware of issues like time constraints, anesthesia problems, ethical issues, limited time, limited space, limited resources etc. So realistic and doable learning objectives should be formed rather than practically impossible ones.* 5. *Objectives should be feasible in all settings of OR for students and faculty in OR. If not feasible, then implementation will be difficult.* 6. *Setting irrational goals can cause depression and frustration.* 7. *if the achievability of an LO is not feasible, students will consider that LO as something out of this world. they will consider it impractical or not related to them, converting the non-feasible Lo into few lines in the curriculum.* 8. *Most important LOs in OR are related to psychomotor skills and to attitudes, so thinking ahead and defining realistically achievable LOs is important for OR teaching.* 9. *Achievable learning objectives should be construed Keeping in mind average students* 10. *Consider the local facilities and logistics, situation, dynamics during surgery* |
|  | 1. Synchronization of the learning objectives with the rest of the teaching. | 1. Synchronization of learning activities, motivates, improves quality of learning and enhances understanding 2. Synchronization helps students to filter unnecessary information and focus on the important aspects | 1. *Two approaches towards a single point are helpful.* 2. *learning in OR is not a separate entity.* 3. *OR experience forms a subset of other skills and knowledge that the learners needs to possess.* 4. *It is the continuation of the whole cycle starting from pathophysiology, intervention (OR/ conservative medications), and recovery. Synchronization will lead to integration of the whole pathway in the students' minds leading to clear concepts and easy understanding.* 5. *All the teaching and learning should be synchronized.* 6. *It will facilitate learning* 7. *Its highly important as this widens the horizon and students can perceive things in a better way and concepts will be more clear* 8. *Topics taught without prior knowledge of the subject can have meagre chances of knowledge retention, if students are informed beforehand about OR list, they can have a better understanding of the procedure* 9. *for example, simultaneously teaching anatomy of thyroid and showing them the surgical anatomy live in OR will help the students to build crisp concepts* 10. *Synchronized study will help lighten the load of less important information and help the majority of students to focus on the high yield points and retain them* |
|  | 1. Importance of Personal Learning Objectives in OR Learning | 1. Personal Learning objectives encourages self-directed learning among students. 2. It shows student’s motivation, preparedness and help them in self-assessment. 3. Personal learning objectives should be realistic 4. Personal learning objectives enable students to improve their weaknesses 5. It should be synchronized to the course objectives to facilitate student learning. 6. Personal learning objectives are more relevant to residents as compared to undergraduate students. 7. Personal learning objectives slow down learning as a group when they are not synchronized to the course objectives. | 1. *Yes, reasonable percentage tends to have their own learning objectives* 2. *Learning should be learner-centered* 3. *It encourages self-directed learning* 4. *it shows their motivation and preparedness* 5. *Every student comes with different aspiration in his mind. Not every mind thinks alike. So, it is inevitable that a lot of the students will have a different notion about how their learning environment is going to come off as. Carrying their own personal goals will help them achieve more and get a good understanding of their objectives as a whole.* 6. *A thing self-learned stays for a longer period of time as it requires brainstorming* 7. *Students know their weaknesses so if they focus on them, can produce better in exam.* 8. *Not sure as it varies for type of student for PG students the personal Los are important, but for undergraduate students personal LOs may not be important.* 9. *But the Los should be oriented to be the minimum achievable goals of what is being taught is at the level of his/her intellect and class level i.e. undergraduate, postgraduate etc.* 10. *Personal LO are important to some extent because students have some questions according to their mental capability that needs to be clarified* 11. *While important to cater to personalized objectives, it is of utmost importance to impart knowledge on a basic level so that majority of students will benefit from sessions. while personalized objectives can be handled in a less formal or overtime setting.* 12. *objectives should be general according to the collective opinion of the students rather than according to the need of the individual. We have fewer resources, so we should find ways to teach the whole batch at once rather than teaching individually* 13. *human mind is complex, and multitasking by the human brain is proven by literature. In addition to the curricular LOs students can have their personal Los, e.g.: learning anesthesia procedures during surgical rotation etc., and its important because they may not find such an opportunity during anesthesia rotation.* 14. *All must have the same learning objective so reach the same goal. With personalized objectives, how would you ensure the necessary acquisition of skills by all graduates. How would you produce standardized graduates? Similarly, having personalized objectives will slow the pace down of the entire session as well as slow down the progression of those who's objectives don't match with the session. Personalized objectives at surgical trainee level make more sense, but at the graduate level, standardized learning objectives to meet the requirements appear more prudent. Here the teacher should take a lead, and the student's input and feedback should guide the process to make it more student-centered.* |
|  | 1. Optimal student-teacher interaction & opportunities for equal participation promote OR learning experience | 1. Smaller class size improves the quality of student-teacher interaction and opportunities for equal participation. 2. Optimal class size favors on-table student-teacher interaction enhancing student’s quality of learning | 1. *Quality of participation depends on the class size* 2. *Individual attention is what most students crave who want to learn. All students should get the opportunity to participate equally* 3. *For me batch size matters a lot; fewer number of students in a batch will help them see the OR activities more clearly, leading to better understanding, vigilance, [and] interaction with the educator.* 4. *Surgeon is already busy in OR, and giving attention to small batch is easy. A small-batch can be allowed to scrub and assist some cases.* 5. *Small batches also do not disrupt the working system of OT assistants and nursing staff thus receive welcoming behavior, that decreases their anxiety in OR [eventually] improving learning* 6. *Allowing Video recording of different aspects of the procedures, letting students scrub up, assist the surgeon and next day follow up classes on the patients which students witnessed in OR can lead to maximized learning* 7. *On table learning builds crisper concepts than classroom learning. A surgeon should make arrangements so that he can explain the anatomy, surgical steps, human body structure while performing surgery. This will build the interests of the students. They will prefer to stay in OR to watch surgery rather than wondering in the corridors and just ensuring their attendance* |
| Educator Related Factors | 1. Interest of educator | 1. Interest of educator motivates students and enhances student participation and hence learning | 1. *A teacher willing to teach, teaches the best.* 2. *His passion ensures optimal educational experience* 3. *Educator's interest is important for any learning experience to be beneficial, and so is OR learning.* 4. *An interested educator is keen to teach and has up to date information and figures. an uninterested educator fails to cater to the needs of students and provides a suboptimal level of information, and fails to capture the attention of students.* 5. *Educators energy gets transferred to the people around him/her. If the educator has high energy as well as interest, it can be contagious to the rest of the session participants.* 6. *Lack of interest can affect students learning negatively* 7. *There is minimal value of 'situated learning' concept in OR. Educator must be enthusiastic enough to show the students the procedure and teach them pinpoint.* 8. *There is no doubt about it, as a teacher being custodian and in charge of that activity plays a pivotal role. So, a teacher taking interest in students learning and perception is the most important factor. I can recall many occasions when I learnt difficult tasks by in time feedback or continuous assistance of my teachers.* 9. *I have seen brilliant surgeons shouting and throwing instruments during the OR procedure. They are great surgeons but never educators or teachers. Similarly, I have seen surgeons who don't want to talk during the procedure. It is highly important that the educator is interested in teaching and, in turn, providing an atmosphere for the students to learn.* |
|  | 1. Importance of educator’s behavior and attitude | 1. Educator’s behavior and attitude facilitates learning in OR 2. The aggressive behavior of educator affects negatively on OR-based learning 3. The educator should have a positive attitude and behavior as the students consider them their role models. 4. Educator needs to be helpful, should show empathy, should be cooperative and non-judgmental. | 1. *Attitude is contagious* 2. *Students idealize teachers and any such behavior can directly impact their learning* 3. *Respect given is respect received. A healthy attitude provides a healthy environment to learn and grow* 4. *Positive and encouraging attitude facilitate students learning* 5. *Behavior and attitude of educator can make students develop interest in that subject or can simply make them hate that subject. Educator's behavior should be influential and he should be approachable for learning even when not in OR because sometimes it is difficult to answer all the queries in one session* 6. *All the participants in the learning session will have a different background of way of learning. It is important that the educator has a nurturing attitude and behavior. Behavior must make it a place of learning and no judgment.* 7. *If teacher is supportive, one can learn difficult tasks easily. Whereas people feel frustrated and lost once they face a hostile attitude and inadequate support of their teachers.* 8. *The teacher’s behavior and attitude not only make the learner more enthusiastic and receptive to learning but also make them keen to teach in the future as well.* 9. *Aggressive behaviors of the teacher may frighten the students. Instead of asking questions, they will try to escape. Learning would become zero. Most surgeons are too aggressive to ask anything even relevant one.* 10. *Rigidity in teaching attitude can lead to reluctance in learning from students and evasion of OR classes altogether, as there is no friendly environment.* 11. *Dealing with your assistants is very important because your students are flowing you* |
|  | 1. Competency of educator | 1. The teacher needs to be experienced. It will help students to achieve their learning objectives and facilitate learning 2. The competent teacher transfers information effectively. 3. Competency is a teaching attitude and is more important than experience. | 1. *Good teachers make good students.* 2. *A good teacher with clear concepts can translate the flow of information in an effective manner.* 3. *The more experienced the teacher is, the more he is capable of inculcating the same in his students. He knows how and when to pass on the basic knowledge and everything that the student needs to know in the best possible manner.* 4. *More competent and experienced, a better motivator for students* 5. *The teachers need to know the topic fully. Saying goes, 'You cannot teach anything to others unless you know them inside out of that topic'* 6. *Experienced teachers know the fine details of the subject and guide students in a proper way* 7. *An experienced teacher will know what the student is feeling or need for learning in OR. there is no match for experience. students can learn at a good pace in the presence of an experienced and competent teacher* 8. *Proper guidance from a teacher sets a student on the right path but over experienced teachers tend to teach above a student's mental capacity* 9. *An experienced teacher is very important as he is aware of the needs and requirements of the students. in my experience teachers with too much experience can be a liability as their teaching methods might have become outdated or too high level for the students.* |
|  | 1. Importance of teaching style | 1. Good teaching style enhances students learning, increased participation and attendance. 2. New teaching strategies improve student’s interest level 3. Teaching style should be friendly, effective and versatile 4. Ongoing commentary during procedure will boost students’ interest and attendance | 1. *A good teaching style makes the subject easier to grasp* 2. *Understanding the way to explain things to students in the OR and the technique to impart knowledge to others is very important. One may know the subject, but if they do not know how to teach others, it will certainly lead to issues.* 3. *Teachers are famous among students due to their teaching styles. teaching style is basically a combination of qualification, experience, competence, personality, and updating according to the needs of the students. teaching style is what conveys everything to the students.* 4. *It is directly proportional to students Attendance in OR* 5. *An interactive and simple teaching style in line with the student’s level and thinking helps information retention.* 6. *a good teacher should simplify the concepts and make it easy rather than imparting a lot of knowledge which the students can’t even contain.* 7. *Different teaching styles can be effective in different types of students.* 8. *While teachers teaching style has some bearing on learning however There is more than one correct teaching style for a given learner, and while ideally, we would like to do to coincide, I think it's important for the learners to be exposed to different styles of teaching as well.* |
|  | 1. Importance of teacher’s preparedness | 1. Preparedness of teacher enhances OR-based student learning. | 1. *Fail to plan, you plan to fail. Teachers preparedness is of utmost importance as they will be the one leading the objectives and must be ready to tackle any queries or confusion that may arise during the session. Failure of preparation can even lead to losing interest and/or respect of the participants involved.* 2. *Preparedness can impart the relevant content in effective manner* 3. *So, If I have read it beforehand and have clear planning, I will definitely share those minor details with my students. This will not only improve my operative outcome, but at the same time will enhance student's learning experience.* |
| Organizational Factors | 1. Significance of OR orientation session. | 1. It provides a guideline about OR protocols and helps students to familiarize with the OR environment. 2. It helps the students to set their learning objectives. 3. Session on OR etiquettes would help students to acclimatize to OR environment | 1. *Orientation to a new environment, especially Operating rooms and expectations of being a responsible health care professional must be conveyed to new students for optimal performance.* 2. *I agree that it's very important for the training to be prepared and should hit the ground running when it comes to an audition. It's a completely new environment for some of them, especially if it is the first time around.* 3. *Most important as orientation to OR norms at the beginning helps the students to familiarize themselves with the environment. A brief overview of likely Los also helps the students to come prepared.* 4. *the students should be given orientation so they can know the rules and regulations of OR so if in case they step out of the lines later, they can be held responsible.* 5. *OR is like a sacred place. It has certain Dos and don’ts. students should be well aware of the precautionary and safety measures and etiquettes of OR.* 6. *Not really, I guess you'll figure it out adventurously* |
|  | 1. Importance of environmental readiness | 1. It improves quality of learning 2. OR should be equipped with all necessary resources such as LCDs and speakers so the students can observe the procedure properly. | 1. *Environmental readiness has a direct impact on student learning.* 2. *The more equipped OR will be, the more opportunities will be for learning.* 3. *e.g., OR should have a large LCD showing live operation. Most of the time, it becomes even hard for the students to see it due to physically inaccessibility.* 4. *This is right. As a fully equipped theatre with sufficient space, minimum noise and temperature regulated, congenial environment is must for better learning experience.* 5. *The role of supportive administration is very important in this regard.* |
|  | 1. Synchronization simulation / Lab activities with OR lessons | 1. It improves OR learning by reinforcing the concepts 2. It will develop student’s interest and polish their skills 3. It will boost the students’ confidence. 4. It cannot replace the real situation; student will fail practically and they will feel intimidated because of lack of practice in a real environment. | 1. *since students cannot experience what they have learned on a patient. simulation programs and lab techniques really help to give them an almost real-life experience.* 2. *Will lead to more concrete understanding about the desired content* 3. *Understanding the OR activities in simulation labs will bring clarity to the students with a hands-on approach, which is not possible in an OR [setting].* 4. *Simulation labs provide students an opportunity to identify their strengths and weaknesses and understand the procedure beforehand* 5. *Shortens learning curve* 6. *It increases the confidence of students before coming to real-life scenarios and should be synchronized.* 7. *Synchronized lab and OR activities may improve learning as what is learnt in OR can be well demonstrated in simulation labs and can be done on models etc. like suturing and likewise other procedures* 8. *This depends on how the participants have grown up in learning. Some who may have never done such a thing will struggle greatly at this and feel intimidated. Even though it [simulation lab] is an excellent tool, it should be given to experts who can relay it in a nurturing way.* |
|  | 1. Importance of adequate visualization in student learning | 1. Better visualization in OR will result in a better understanding of the anatomy and operative concepts. 2. Equipment would improve visualization in OR such as LCDs etc. 3D visualization is important for OR-based learning. | 1. *1st level of skill is to observe. For observation good vision of the surgical field is mandatory* 2. *visual memory is stronger than reading* 3. *3 D visualization of operative field anatomy is important, and efforts should be made to enable students to visualize this.* 4. *Without clear visualization the students can’t understand the procedure at hand and will be left to their imagination about that is going on in the operative field* 5. *Seeing things in real life rather than from books clarifies many ambiguities* 6. *OR should have a large LCD showing live operation. Most of the time it becomes even hard for the students to see it due to physically inaccessibility* 7. *If some students miss the procedure one time, they can be shown the video of the same procedure, or they can visualize this next time as the clinical cases repeat frequently in OR.* |
| Psychosocial factors | 1. Impact of anxiety in OR environment | 1. Anxiety slows down students learning and distracts students and teachers. 2. The aggressive behavior of the surgeon induces anxiety in OR 3. The students need to learn how to adjust to the OR environment. | 1. *Congenial environment is important for learning* 2. *Anxious and tense situations tend to dampen the minds absorptive potential and lead to loss of focus.* 3. *Anxiety can affect students learning negatively* 4. *But teacher can help to control it* 5. *Most students are excited than anxious. a tour of OR on the first day by a senior teacher can help a lot.* 6. *We must prepare them before going to the theater and explain what is going to happen there so that they may not get suddenly exposed and get anxious or upset* 7. *Anxious and angry surgeons and teachers make the students frightened and decrease the learning drive.* 8. *Very important phenomenon as dealing with a human life so they should know that their teachers are also stressed.* 9. *Mental state has an enormous impact on the students learning. If the student does not feel comfortable or is intimated, they would be occupied with trying to avoid any miss-step instead of learning.* 10. *As OR environment is unfamiliar in addition to multiple intrinsic and extrinsic factors. The overall environment in a busy theater creates a lot of anxiety in students, and it can affect their performance as well.* |
|  | 1. Effect of fear and intimidation in OR learning environment | 1. Fear hampers learning while positive OR environment facilitates learning. 2. Fear of being criticized is common among students 3. Fear results in missing sessions, low participation, lack of interest, poor self-esteem, and demotivation. 4. Fear can be managed by encouragement and training 5. The orientation session reduces fear among students | 1. *Fear and intimidation will only lead to avoidance behavior and lack of confidence in the students.* 2. *It will not only impede the learning process but also diverts student’s attention.* 3. *Fear of being criticized is quite common* 4. *Fear has a negative impact and has to be overcome to learn in OR.* 5. *Intimidation or fear will make student not participate and learn things* 6. *It only happens if the environment is not friendly. e.g. surgeon/educator is very aggressive* 7. *Most people take fear and intimidation as a negative emotion and hence should be avoided to replace with a respectable and safe learning environment where the student would want to return over and over.* 8. *Teacher who use intimidation and fear or think that either of this can or may be used as an instrument of teaching should be banned from operating!* |
|  | 1. Impact of feeling welcome in OR | 1. Welcoming students in OR will increase attendance, participation, interest and make them feel part of the team 2. Teachers use aggression as a teaching strategy which should not be practiced. 3. The surgeons should perceive their students as future doctors and should train them well. | 1. *Having a positive emotion attached with not only improve motivation but will improve attendance and capacity to learn.* 2. *Welcoming creates a positive environment and positivity no doubt affects learning in OR in a healthy way* 3. *beginners should be encouraged rather than insulted for not knowing. Theoretical knowledge is completely diff than the practical.one learns from the start when he/she starts practical work.* 4. *This is basic human psyche that they need to feel welcome in the environment that they are fear, intimidation and bullying /harassment /anxiety none of those work and should not be used and cannot be justified to be used as a tool for teaching.* 5. *Students are insulted for not answering the questions bullied and harassed by surgeons* |
|  | 1. Impact of victimization in OR environment | 1. Victimization as tool for learning should not be allowed 2. Victimization of the students will result in, low attendance, demotivation, low participation, fear, loss of interest and excitement | 1. *OR should be a nonbiased sanctity, supporting anyone and everyone who comes to the OR* 2. *Feeling of unwanted or being abused completely drains the students interest not only in surgery but in the entire field of medicine. Safe environment for all involved is a must.* 3. *Will lead to demotivation and disinterest* 4. *The students will not attend the class to avoid the victimization* 5. *Staff members should be highly discouraged and refrained in OR to taunt students that are here for learning purposes.* 6. *Staff members behavior can impact learning in OR. Healthy environment makes learning effective* 7. *Victimization will lead to loss of communication and fear in students, which is detrimental to a good teaching environment.* 8. *Negative experience affects future self-efficacy* |
|  | 1. Impact of self confidence | 1. It is a positive factor that improves quality of learning 2. Confident students learn better, show good social skills, and participate more. 3. Nonjudgmental teaching style improves student’s confidence 4. Overconfidence affects negatively | 1. *Self-confidence is important for any learning experience to be beneficial so is OR learning* 2. *Self-confidence is important, but there is a demarcation between self-confidence and overconfidence. Many students develop confidence after a few sessions. Once you are confident you actually participate actively. Self-confidence has no more importance other than this* 3. *OR learning is a type of opportunistic learning. students with high self-confidence often get the opportunity to scrub with consultants and assist leading to increase interest, respect among colleagues, and more self-confidence.* 4. *students with high self-confidence are not reluctant to go to new environments like OR and also do not fear or get intimidated by OR staff* 5. *Gestures to boost their morale help them control their anxiety. It prepares them to face the worst scenarios and improves their memory and retention.* 6. *It is important to deal the serious intraoperative complications during surgery* 7. *When a student is inculcated with self-confidence, he is more than willing to put in his heart and soul into the subject. He knows he can perform well when he is confident. Shattering his confidence then and there will ruin his self-esteem and might not make him able to learn anything at all.* |
|  | 1. Impact of student motivation | 1. Motivation increases student attendance, helps them to achieve their learning objectives 2. Positive reinforcement increases motivation 3. Self-motivation is more important than the external motivation 4. The teachers are responsible to motivate their students | 1. *positive reward builds motivation and that builds drive for learning.* 2. *Motivation drives learning* 3. *Motivation means eager to learn from mistakes, more experiences to learn are required to achieve competency* 4. *Motivation has a direct impact on the ability of learning and effort that the student will put in each learning session.* 5. *Those well motivated will gain more. An environment needs to be offered so that all feel part of it.* 6. *Improved Attendance n subsequently learning* 7. *The environment at OR is intimidating, new and critical. it has its own feel. only those who are motivated enough to continue in the challenging environment learn the most* 8. *Inner or self-motivation is more important than external motivation, but yes at time external motivation also plays an important role* 9. *The more you motivate a student, the more confident he becomes and not to mention the more interested in the subject. So, a bit of motivation every now and then will help him dramatically.* |
| Student Related Factors | 1. Student’s Prior Knowledge | 1. Student’s prior knowledge and skills can be really beneficial in OR learning. | 1. *Simple saying ‘Eyes can't see what the brain doesn't know’* 2. *A well orientated student can grasp more information and skills than an uninformed student* 3. *Prior knowledge and orientation are always helpful in building new knowledge, follow[ing] the constructionist philosophy* 4. *Having some theoretical knowledge of the task to be performed gives students the foundation to build on during the practical procedure.* 5. *The teacher should elicit the current status of student knowledge and then build from there* 6. *Pre-existing knowledge about a certain topic can augment the understanding of the student* 7. *Prior knowledge leads to solid understanding rather than just observation* 8. *"eyes can see what the mind knows" if a student attends an OR session without the prior knowledge of sterilization, he [or she] cannot comprehend the importance of sterilization and will disturb the sterilization of OR. If a student comes to OR without knowing anatomy of an area, he cannot get the true essence of the surgical procedure.* 9. *In some instances, it may help in understanding; however, it may also give them a false sense of security and lose of focus as they may think they already know the answers.* |
|  | 1. Student’s Pre-lesson self-review of reading material | 1. It facilitates learning new concepts and better understanding 2. prior knowledge supports further gain in knowledge | 1. *Its important that the students go through the learning material beforehand to enhance their learning.* 2. *students self-review the pertinent material with the intention of learning and looking forward to go to OR and learn more there. so just reviewing the material shows the intention of learning and self-regulation. secondly it may lead to the rise of some questions in the student's mind that he can get answers to directly in OR, which improves learning* 3. *Most students look for a more hands-on approach when it comes to activities in the OT. Interest level towards learning material is low.* 4. *As more focus should be on the practical learning in OR .so this factor becomes less important.* 5. *pertinent material when read pre-lesson, leads to more cognitive retention* 6. *It helps them to set their learning objectives* 7. *It enhances a better understanding of the concepts of OR-based learning of the students.* 8. *It is directly related to self-motivation.* 9. *Student can identify their weak areas to improve them* 10. *It is not important as the focus is on practical learning* 11. *Students should watch videos before OR procedure[s]* 12. *This can be done through providing manuals and study guides prior to introduction of OR environment* 13. *students self-review the pertinent material with the intention of learning and looking forward to go to OR and learn more there. So just reviewing the material shows [their] intention of learning and self-regulation.* 14. *secondly it may lead to the rise of some questions in the student's mind that he can get answers to directly in OR, which improves learning* 15. *Most students look for a more hands-on approach when it comes to activities in the OT. Interest level towards learning material is low* |
|  | 1. Student’s Readiness to participate | 1. Self-regulation and self-readiness determine the student's perceptiveness to learning | 1. *Students should sleep well and [should be] medically fit* 2. *The student s should [exhibit] readiness to participate and assist* 3. *Self-regulation and self-readiness determine the student's perceptiveness to gain [in] knowledge [and learning]* 4. *It facilitates goal-oriented study.* 5. *It enhances student’s learning and better understanding of the clinical concepts.* 6. *It facilitates adjustment of students in the OR environment* 7. *Students' readiness to participate and to self-regulate their learning helps the educator facilitate students' learning by tailoring the session to their skill levels and needs.* |
|  | 1. Student’s Focus on Practice | 1. Repetition of the skills is mandatory for improved cognitive, psychomotor, and affective retention. 2. Objective assessment of psychomotor skills can improve the focus of students | 1. *Skills need repetition for improvement* 2. *Repetition of the skills is mandatory for cognitive, psychomotor, and affective retention.* 3. *focus on newly acquired skills can provoke interest for discovering and learning more in students, thus may help in improved learning* 4. *This is less important for generic skills and more important for specific skills. As ‘practice makes perfect’ so, practice of newly acquired skill is very important if that skill is to be incorporated into the student's current skill set.* 5. *objective assessment of one's performance and review help with deliberate practice and learning.* |
